# Haplotype-Based Approach Represents Locus Specificity in the Genomic Diversification Process in Humans (*Homo sapiens*)

**DOI:** 10.3390/genes15121554

**Published:** 2024-11-29

**Authors:** Makoto K. Shimada, Tsunetoshi Nishida

**Affiliations:** Center for Medical Science, Fujita Health University, Toyoake 470-1192, Aichi, Japan

**Keywords:** extended haplotype homozygosity (EHH), S* analysis, introgression, incomplete lineage sorting (ILS), ancient

## Abstract

Background/Objectives: Recent progress in evolutionary genomics on human (*Homo sapiens*) populations has revealed complex demographic events and genomic changes. These include population expansion with complicated migration, substantial population structure, and ancient introgression from other hominins, as well as human characteristics selections. Nevertheless, the genomic regions in which such evolutionary events took place have remained unclear. Methods: Here, we focused on eight loci containing the haplotypes that were previously presented as atypical for the mutation pattern in sequence and/or geographic distribution pattern with the model of recent African origin, which constitute two major clusters: African only, and global. This was the consensus model before information regarding introgression from Neanderthal (*Homo neanderthalensis*) was available. We compared diversity in identical datasets of the modern human population genome, with the 1000 Genomes project among them. Results/Conclusions: This study identified representative genomic regions that show traces of various demographic events and genomic changes that modern humans have undergone by categorizing the relationships in sequence similarity and in worldwide geographic distribution among haplotypes.

## 1. Introduction

The history of human genetic polymorphism research is a good example of the frequent updating of genomic science technology as well as demographic models of gene flow (For review of Genetic Techniques [1]). The relationship between the genomic diversity among African and non-African haplotypes represented in mitochondrial DNA (mtDNA) gene trees constituted a pivotal support for the recent out-of-Africa (OOA) model of human origin, which had been the subject of considerable debate regarding the origin of modern humans. The topology of the gene tree with deep bifurcation of one cluster composed of only African haplotypes and other cluster of African and non-African haplotypes showed a single origin of modern humans (*Homo sapiens*). Furthermore, less variation of non-African than African represented a bottleneck effect at the out-of-African event [2]. This first mtDNA gene tree was obtained by the method called restriction fragment length polymorphism (RFLP) [2]. Subsequently, the same tree shape was also obtained regarding modern human populations with a branched Neanderthal sequence outside of the variation of modern humans using a comparison of the mtDNA sequence [3]. This allowed the OOA model to prevail regarding the origin of modern humans. At that time, the prevailing perspective was that one of the two primary models, the OOA model or the multiregional model, was the correct one. Consequently, there was a period when models based on other ideas were not dominant. In fact, since the era of exploratory nuclear genome research, there have been gene fragments that deviate from the tree-topological pattern of the OOA model. Since the utilization of nuclear genomic regions as markers in population evolutionary studies commenced, a number of gene trees for specific nuclear genome regions have been reported as exhibiting discrepancies from the OOA model, which represented the shape of mtDNA gene trees [4,5,6]. The major explanation for these data at that time was an ancient introgression post out-of-Africa, or admixtures with archaic human groups, such as Neanderthals and Asian *Homo erectus*, which was one of the first explanations that came to mind for many researchers [7,8,9,10,11,12,13]. In addition to introgression and admixture, other factors may contribute to the observed discrepancies in the gene tree topology predicted by the OOA model. In contrast to the mitochondrial genome, the nuclear genome is a recombinant assembly of fragments with disparate functional constraints and a divergent evolutionary history, resulting in incompatibilities between the gene tree and the population tree. Moreover, human populations contain heterogeneous individuals with different genomic signals of frequent genomic changes such as inversions, duplications, and insertions, as well as recombination between haplotypes encountered by introgression, resulting in deviation of gene tree topology from the OOA model. Thus, the progress of knowledge of genomics on human populations revealed that the demographic history of human populations has a more complex background than when the mtDNA gene tree was published. Conversely, given that the demographic history of the human species is more complex than the mtDNA gene tree indicated, there is no extensive knowledge of which genome fragments bear the traces of each evolutionary event and in what shape of the gene tree.

Gene tree shapes are the indices useful for predicting various evolutionary events in addition to the OOA model supported by mtDNA. For instance, a gene tree with numerous branching events occurring within a relatively short timeframe is indicative of explosive evolution. Furthermore, gene duplication events that occurred prior to speciation can be identified by a gene tree that demonstrates a closer relation between orthologues of other species than between the duplicated genes (paralogues) of the same species [14,15]. Two pivotal considerations when interpreting genomic data to elucidate population history are as follows:

1. The suitability of the target locus as an indicator of historical processes.

2. The adequacy of the assumption that population history is characterized by differentiation and disappearance as a basis for explanation. History thus far has been one of accumulation, with each subsequent issue becoming more apparent as the research progresses and the actual situation becomes clearer. By applying consistent procedures for haplotype comparison across these nuclear genomic regions, we have identified representative genomic regions that appear to have experienced various demographic events and genomic alterations (genome translocations) that are characteristic of modern humans. As a result, inclusive insights were obtained into the patterns of sequence similarity and geographic relationships among haplotypes in each of these segments. The pattern that unquestionably demonstrated introgression was the clustering of haplotypes from archaic hominins, specifically Neanderthals or Denisovans, in modern non-African humans in the gene trees. Additionally, our S* analysis provided further evidence of alignment between the introgression scenario and the data regarding the geographic distribution of haplotypes, thereby reinforcing the notion of introgression without contradiction. These include the 30.9 kb region of 12q24.13 (OAS locus) and the 176.9 kb region of 3p21.31 (HYAL locus) in our analysis. Other loci that we have included illustrate instances that diverge from the mtDNA tree topology, not as a consequence of typical introgression processes, but rather as a result of the influence of selection and recombination, respectively. Thus, this research offers representative examples of introgression from archaic to modern human populations, as well as patterns of gene trees that deviate from the OOA model in various genome fragments. These illustrative instances will prove useful for further evolutionary study, as they facilitate the inference of demographic events.

## 2. Results

### 2.1. Target Genomic Regions (Loci)

To illustrate diversified haplotypes in modern human populations that arose due to various factors, we searched the literature for reports that discussed the relationship of highly diverged haplotypes with the OOA model (see Section 5 for detailed criteria). We selected diverged haplotypes of eight genomic regions based on the traceability of genome coordinates published before 2013. A focal SNP was determined in the most diverged haplotype for each locus (Table 1). Then, we defined the locus as a region under LD by extracting SNPs closely related (i.e., *r*^2^ ≥ 0.8) to the focal SNP (see Section 5 for details). The length of the target genomic region ranged from 23,198 bp (RRM2P4) to 550,655 bp (17q21inv) (Table 1). The nomenclature of the loci was derived from the original papers, but the genomic regions utilized for analysis in this study may encompass multiple genes following LD extension.

### 2.2. Phylogeny of Haplotypes

**Figure 1:**
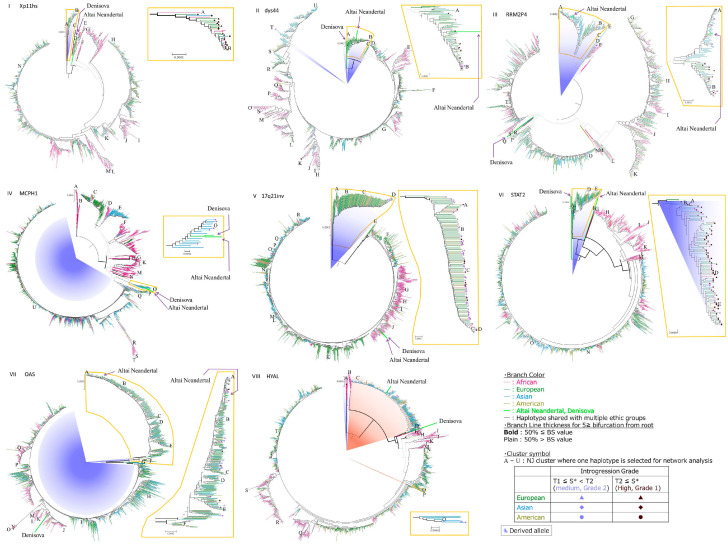
Tree_Panel. NJ trees for haplotypes of modern humans and archaic hominins (Altai Neanderthals and Denisovans) of eight representative loci. (**I**): Xp11hs, (**II**): dys44, (**III**): RRM2P4, (**IV**): MCPH1, (**V**): 17q21inv, (**VI**): STAT2, (**VII**): OAS, (**VIII**): HYAL. Sample origins of haplotypes are expressed by colors of branch. Haplotypes of archaic hominins and clusters shared across multiple continents are indicated by thick light green branches and thin black lines, respectively. Line thickness of branches within five bifurcations from the root indicates two classes of bootstrap values of the downward clusters [i.e., <50% (thin) and ≥50% (thick)]. Haplotypes with introgression grades defined by S* analysis are marked by dark red (high) and pale blue (intermediate). Clusters where one representative haplotype was selected for network analysis are shown in capital letters. Derived allele distributions of focal SNPs in representative haplotypes are depicted by a blue background. When the focal SNP differs from the SNP representing an unusually diverged haplotype reported by the original study, the distribution of the focal SNP from the original study is shown in a brown background (see Section 5).

**Figure 2:**
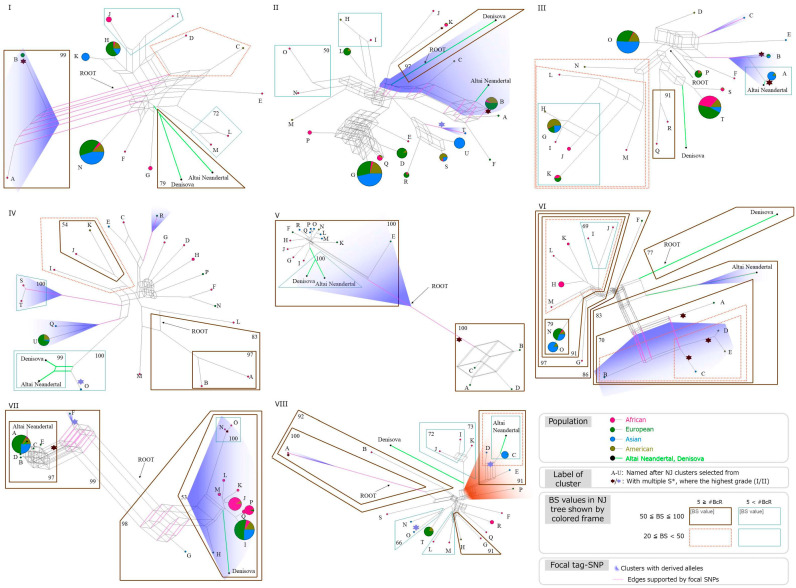
NetworksPub4JHq. Phylogenetic network of major haplotypes representing major phylogenetic clusters for eight loci. (**I**): Xp11hs, (**II**): dys44, (**III**): RRM2P4, (**IV**): MCPH1, (**V**): 17q21inv, (**VI**): STAT2, (**VII**): OAS, (**VIII**): HYAL. These haplotypes were selected from major clusters of the NJ tree to avoid bias in each locus (see Section 5 for details). Sample origins of haplotypes are expressed by colors of branch tips. The color and thickness of frames surrounding haplotypes indicate bootstrap values and distances (i.e., number of bifurcations from the root point) of the clusters in the NJ trees, respectively. Distributions of derived alleles of focal SNPs and edges bearing focal SNPs are depicted by the blue area and pink line, respectively. Hexagrams indicate clusters containing haplotypes with introgression grades defined by S* as in Figure 1. Color of the hexagrams are determined by the highest S* in each cluster. Small hexagrams indicate African clusters containing haplotypes with S* in admixed American samples.

We constructed a distance-method-based phylogenetic tree (i.e., neighbor-joining, NJ) to assign all haplotypes into clusters (Figure 1). Then, we selected a representative of each cluster uniformly based on criteria that we established. To represent other possible relationships among the clusters, we constructed a phylogenetic network (Figure 2). To discuss the processes by which human genome diversity develops, such as population subdivision and gene flow, we classified the topological patterns of haplotype trees (Table S1; Figure S1). We defined typical OOA patterns as African haplotypes and non-African haplotypes nested within African haplotypes, considering the bottleneck effect of OOA under the assumption that mutations occurring after the OOA event are too few to affect tree topology (Figure S1). We found the typical OOA pattern at all loci examined, including multiple nested patterns found at dys44 and HYAL loci (Table 2). We also found that both Altai Neanderthals and Denisovans were clustered together for three loci, Xp11hs, MCPH1, 17q21inv, within the obtained haplogroup phylogenies, which does not necessarily reflect introgression of both archaic humans (Table 2).

### 2.3. S*

S* is a method of estimating the presence or amount of gene flow between subpopulations by detecting combinations of rare alleles [26,27]. We performed S* analysis to estimate distinct gene flow events from archaic hominins after OOA using Africans as a reference population and to detect novel SNP allele combinations in modern humans that only exist in Eurasia. We modified S* analysis to apply phased genome sequence data and highlight haplotypes with high S* scores. Then, we classified the obtained values of S* into three classes (i.e., high, intermediate, and low introgression grades; see “S* analysis”; “Algorithm” in Section 5). The S* score showed signs of gene flow after OOA at all eight examined loci to a greater or lesser degree (Table 2; Figure S2).

We observed clusters that included both haplotypes with high S* scores and haplotypes of known archaic hominins (i.e., Altai Neanderthals and/or Denisovans) in clusters *A* and *B* at dys44, clusters *A* and *B* at RRM2P4, cluster *O* at MCPH1, clusters *A* to *F* at OAS, and clusters *C* at HYAL (Table 3, Figure 1). These findings indicate the possibility of gene flow between the archaic hominins and Eurasians after OOA. Three of the loci (dys44, RRM2P4, and OAS) showed topological patterns of haplotypes (outer cluster composed of various populations, cosmopolitan, with high S* and Neanderthals, type FE; Figure S1) that likely result from introgression of Neanderthals in Eurasia after OOA.

Although clusters composed of archaic hominins and Eurasians also had high S* scores in cluster *O* of MCPH1 and cluster *C* of HYAL, these topologies can be explained by ancient subdivision within Africa before the Neanderthals left the continent or gene flow in the opposite direction to the introgression in Eurasia (Type Af; Figure S1).

In the gene trees of Xp11hs and STAT2, we also observed S* haplotypes among modern humans in the outermost clusters that did not contain the haplotypes of known archaic hominins (Figure 1, Table 2). These diverged clusters of Xp11hs and STAT2 contained high-S* haplotypes composed of various populations (cosmopolitan clusters) without geographical aggregation (Table 3). Generally, a random geographical distribution is considered to represent ILS [28], and these diverged clusters at these two loci can be attributed to events that produce polymorphisms that existed before or during OOA, rather than introgression from archaic humans after OOA. Although a similar pattern was also shown at the 17q21inv locus, introgression was not necessarily needed to explain this pattern because of limited recombination between chromosomes, with different orientations caused by inversions (see Section 3).

Some of these phylogenetic trees and networks showed the effect of recent admixture in Americans because American samples used in the 1000 Genomes Project are from “admixed” populations of Africans, Europeans, and Native Americans collected at various sites in North America, but not Native Americans. This is documented as “Admixed American (AMR)” in The International Genome Sample Resource [29] (Table S2). Two American haplotypes with high S* scores were observed in African clusters (cluster *A* at HYAL and cluster *N* at OAS). Both African clusters were small and separated from other African clusters in the tree (individual ID and population of haplotypes in clusters *A* and *B* of the HYAL locus can be found in Tables S3 and S4). These findings indicate that Americans inherited these haplotypes from Africans, and these haplotypes are rare even in Africans, resulting in their high and intermediate S* scores.

### 2.4. Extended Haplotype Homozygosity

Extended haplotype homozygosity (EHH) is a measure to detect recent positive selection based on decay of haplotype identity as a function of distance from the focal SNP site. Sabeti et al. (2002) originally developed the EHH statistic to detect positively selected alleles by focusing on the relationship between an allele’s frequency and the extent of LD surrounding it [30]. That is, under neutral evolution, a more recently derived allele requires a long time to reach a high frequency, by which time the LD around it will have decayed considerably. Under positive selection, however, a derived allele rapidly increases in frequency with the retention of LD [30].

**Figure 3:**
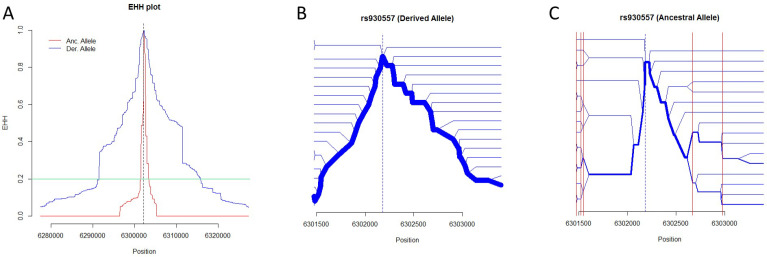
EHH analysis for MCPH1. (**A**) EHH plot of MCPH1. EHH is plotted in the genomic region showing EHH < 0.05 in at least one allele. Red and blue lines indicate EHH for ancestral (Anc) and derived (Der) alleles, respectively. The bifurcation graphs were generated within the region showing EHH > 0.2 (aqua green line) in both alleles. (**B**,**C**) Bifurcation graphs for the MCPH1 locus. The position of the focal SNP site is shown by blue dotted lines. The width of blue lines represents the frequency of haplotypes bearing derived (**B**) and ancestral (**C**) alleles of each focal SNP. Red lines in (**C**) indicate SNP positions that make bifurcations at multiple branches. EHH analysis of other loci is shown in Figure S4.

Our EHH analysis indicated selection on only one specific allele at the MCPH1 locus. The EHH range of the derived allele “*C*” from rs930557 was longer than that of the ancestral one “*G*”, which is explained by the occurrence of a selective sweep at the MCPH1 locus (Figure 3A). The MCPH1 bifurcation graph showed that almost all bifurcations were observed in a single lineage of haplotypes with the derived allele “*C*” at rs930557, which suggests that a novel mutation generated a novel bifurcation (Figure 3B). This is explained by a selective sweep of haplotypes bearing the derived allele “*C*”. In contrast, that for ancestral allele “*G*” showed a succession of bifurcations in multiple branches at common genomic positions, such as 6301472, which indicates the existence of SNP sites that share alleles with other haplotypes (Figure 3C; Figure S3). This indicates the occurrence of recombination among haplotypes bearing the ancestral allele “*G*” at rs930557 (Figure 3C).

## 3. Discussion

We reconstructed the phylogenetic relationship among haplotypes of 1092 individuals from 14 modern human populations with archaic hominins at eight loci that had been reported as unusually diversified before publication of the first archaic hominin genome. This study demonstrated that each locus has its own evolutionary history, including selective sweep, partial reproductive isolation resulting from inversion, and ILS, as well as introgression from archaic *Homo*. Based on these results, we showed representative examples of (a) introgression from known archaic hominins such as Neanderthal and/or Denisova, (b) selection effect, and (c) recombination effect for deviation of gene tree shape of OOA model.

### 3.1. Several Patterns of Introgression Diversified the H. sapiens Population

The three example loci that likely represent introgression from known archaic hominins are dys44, RRM2P4, and OAS (Figure 1, Table 2). This is based on tree topology showing the outside cluster that contains the ancient haplotypes (i.e., Neanderthal, N; Denisovan, D) and the Eurasian (E) or Cosmopolitan (C) haplotypes with high S* scores, ((N or D), (E or C)) in Newick format (Table 2). By locating these clusters at the outermost part of each tree’s topology (topology pattern FE; Figure S1), these findings can be explained by introgression from known archaic hominins assuming a conventional population tree without ILS, i.e., (archaic, (South African, (East African, Eurasian))).

The comparison of haplotype tree topology among loci indicates gene flow events other than introgression from known archaic hominins as per the following examples. The haplotype tree of the MCPH1 gene showed a cluster containing haplotypes of Asians with high S*, Neanderthals, and Denisovans (cluster *O*). Contrary to the findings for the three loci mentioned above, cluster *O* was not located at the outermost position (see the Section 3.6. “Comparison Between Phylogenetic Inferences by Network and Distance Methods”). This requires other explanations than gene flow from archaic to modern humans. One possible explanation is reverse gene flow under the ancient subdivision within the African continent, in which there was an ancient subdivision within Africa before the Neanderthals left the continent. This ancient subdivision model assumes an ancient population structure that persisted before divergence of the ancestral Neanderthal population until OOA of modern humans [7,31]. Although this model is not supported by previous studies [31,32,33], this possibility requires further empirical studies on loci showing this pattern. Another possibility of gene flow is that from unknown (i.e., unsequenced) archaic hominins. In contrast to the three loci mentioned above, the haplotype tree of STAT2 indicates a high S* score at the basally diverged Eurasian lineage, which suggests introgression from unknown archaic hominins in Eurasia (Ea; Figure S1). Although the possibility of other scenarios, such as insufficient sample sizes of the reference population or novel combinations of rare alleles by recent recombination, cannot be entirely ruled out in this type of topology, an earlier study showed several gene flow events among archaic human groups, which included unknown archaic groups (i.e., not Neanderthals and Denisovans) [11].

Our haplotype tree comparison may suggest a difference in breeding history between Neanderthals and Denisovans. Although the Neanderthal and Denisovan haplotypes had different locations in the tree topology of three loci (dys44, RRM2P4, OAS), the Neanderthal haplotypes showed the possibility of introgression based on tree topology, but those of Denisovans did not show a clear trend (Table 3). This difference between Neanderthal and Denisovan haplotypes can be explained by a history of migration and hybridization with modern humans. Notably, Denisovan genomes contain components that were introgressed from other archaic populations, which were deeply diverged from the common ancestor of Neanderthals, Denisovans, and modern humans [11]. This may explain why Denisovan genomic fragments with various backgrounds present more varied locations in the gene trees based on these loci than those of Neanderthals, although this possibility requires further study.

### 3.2. ILS of Ancestral Polymorphisms

ILS of ancestral polymorphisms is a phenomenon in which ancestral gene copies that existed and started to diversify before population/species separation represent a gene genealogy that differs from the population/species phylogeny (see [7,28,34] for detailed discussions of the distinction between ILS and introgression; Table S5). The most outstanding example representing ILS of ancient polymorphism is at the Xp11hs locus. In the tree based on this locus, the outermost two clusters (A and B) contain African and Eurasian haplotypes with high S* scores (Figure 1). Considering the S* concept, this clustering is aberrant because Africans are the reference population. This might be explained by these five African haplotypes representing a small proportion of all 377 African haplotypes used as a reference in S* calculation (Tables S3 and S4). It is assumed that Eurasians must be a subpopulation of Africans (a reference population) under the OOA model; this means that all original or closely related haplotypes of the OOA population (Eurasian) should remain in Africa today and be included in the reference population of S* analysis given the vast sampling with a sufficient sample size of Africans. If these conditions are not fulfilled, such as there being a disproportionate distribution of rare alleles, a false-positive S* signal might occur. Rare haplotypes that existed in East Africa via ILS before OOA may show a false high S* score if they were included in the OOA migrating population but not included in the reference population used in the S* analysis. The individuals with the five African haplotypes included both East and West Africans (Tables S3 and S4), which may indicate that ancient polymorphisms persisted before the subdivision between East and West Africans.

Similarly, the H2 haplotype at the 17q21inv locus contains both African and high-S*-scoring Eurasian haplotypes (Figure 1). This is also due to the disproportionate distribution of rare haplotypes between reference and migrated populations; there was no sampling of H2 Africans in the reference population despite high colocalization of H1 and H2 within Eurasia.

Therefore, S* analysis based on gene trees is a valuable tool for distinguishing between ILS and introgression, at least in some cases. Nevertheless, this research indicates that researchers must exercise caution when selecting reference populations. Insufficient sampling from African populations and/or the extinction of ancestral African populations can result in false-positive S* scoring, which may be particularly prevalent when there was a highly diverged population structure prior to OOA. Further simulation-based studies that focus on these two scenarios are necessary for this tree type.

### 3.3. Partial Reproductive Isolation Resulting from Inversion (Polymorphic Inversion)

We selected 17q21inv to demonstrate an effect of chromosomal rearrangement on population structure and gene flow by its unique features, as suggested in accumulated previous studies. Our investigation of 17q21inv suggests that gene flow from archaic hominins is not necessarily needed to explain the obtained tree topology with deep branches. The most obvious reason for this is that the Neanderthal and Denisovan haplotypes are included in the African cluster, which does not fit the scenario of introgression occurring outside of Africa. Historically, this inversion was found in a region where recombination was not observed, namely, a region of around 2 Mb in 17q21.31 that is accompanied by segmental duplications (SDs) [35,36,37,38]. SDs played a critical role in chromosomal rearrangement during primate evolution [39] (e.g., [40]). Indeed, it has been shown that chromosomal rearrangements occurred frequently and repeatedly in this region during primate evolution [41]. Accordingly, the ancestral haplotype of this inversion is still under debate [42,43]. The 17q21inv polymorphism is specific to the human lineage [41]. Baker et al. (1999) [44] named the common (non-inverted) haplotype “H1” and the rare (inverted) haplotype “H2”. Steinberg et al. (2012) [42] suggested that the inverted orientation (H2 haplotype) was the ancestral state of the *Homo* lineage, which was also confirmed by our rooted tree (Figure 1). The H1 haplotype emerged by (re-)inversion approximately 2.3 million years ago (Mya) and became dominant. This predominance of the H1 haplotype was clarified by the observation of this haplotype in Neanderthal [7] and Denisovan genomes [45], which was also reinforced by our results.

Copy number polymorphisms of the SDs arose in the H1 and H2 lineages around 250,000 years ago and 1.3 Mya, respectively. An H2 haplotype bearing SD (H2D) rapidly increased in frequency by up to 10–25% in Europe, resulting in very little variation within H2D [42]. The cause of this rapid increase in frequency of H2D—whether intense positive selection or rapid population growth—is still unclear, although positive selection was assumed in previous studies [19]. Alves et al. (2015) [46] demonstrated negative trends of Tajima’s D [47] in both H1 and H2 lineages, although H1 is more variable than H2 in terms of nucleotide diversity. This deviation from neutrality for both the H1 and H2 lineages challenges the possibility of a selective sweep on H2, although the authors were careful to indicate that this was only speculation. Generally, a demographic event does not affect only a single locus, which refutes the possibility of rapid population growth. Our EHH analysis does not suggest a selective sweep in the H2 lineage or any notable difference between the two haplotypes. Considering restrictions in recombination between inverted and non-inverted haplotype families, the negative trends of Tajima’s D can be explained by each haplotype family acting like a genetic barrier, which divided haplotypes and resulted in a smaller effective population size and longer LD than for other loci. This is a similar phenomenon shown in a population just after admixture of two divided populations.

Two studies evaluated population genomics using SNP genotype data from worldwide populations focused on the H1 [42] and H2 [46] haplotype families. They found that these haplotypes independently had African clusters that diverged first within each haplotype family. This indicates that both H1 and H2 haplotype families existed within Africa before OOA of modern humans (*H. sapiens*). The limited representation of African samples in the phylogenetic analysis of our study may account for the absence of a cluster comprising exclusively African individuals, as observed in the H2 haplotype family (haplotypes A–D). Taking the obtained findings together, our study confirmed these previous findings, indicating that ancestral polymorphisms were maintained before the divergence of modern humans from ancestral Neanderthals and Denisovans. Our study indicated that the current distribution of H2 haplotypes does not need to be explained by contact between modern Eurasians and Neanderthals and/or Denisovans. Additionally, we suggest that introgression from other unknown archaic hominins is not necessarily required to explain the haplotype distributions at the 17q21inv locus. It is therefore more probable that long-lasting ancestral polymorphisms with restricted recombination between the two haplotype families resulted in the phylogeny with two distinct clusters. However, the basal bifurcated cluster (H2 cluster) consisted only of Eurasian samples, which may be attributed to the loss of haplotype variation from Africa and archaic hominins or insufficient sampling.

### 3.4. Effects of Selection

The best example representing the effect of selection on the pattern of haplotype diversification is the MCPH1 locus. Our EHH using pooled data did not suggest the occurrence of elongated LD at any other locus examined except MCPH1, indicating that there was no similar level of influence of positive selection on the modern human population at the other loci examined [allelic selection (AS) in Table 2]. Although Stefansson (2005) [19] obtained evidence suggestive of positive selection in the H2 lineage at the 17q21inv locus, this was not detected by our EHH with pooled data (Table 2). This confirmed the result obtained using the ln(Rsb) statistic that determined the occurrence of selection by comparing EHH for an SNP between two populations [48].

We also noted that the bifurcation graphs depicted a skewed distribution of branching points of haplotypes in OAS, HYAL, and Xp11hs (Figure S4). Consequently, the numbers of haplotypes did not increase in proportion to the distance from the focal SNP position in these regions. This may have been due to a change in SNP density, which represents the existence of differences in evolutionary constraints within the high-EHH region; the region with fewer SNP sites was confirmed to overlap with the promoter region of the SHROOM4 gene in Xp11hs, the transcribed region of the OAS1 gene, and the transcribed region of the HYAL3 gene [constrained region (CR) in Table 2]. The presence of fewer SNP sites within this region is explained by lower maintenance of new mutations as SNPs due to a low rate of fixation of new mutations.

Caution should be exercised when subjecting rare alleles to EHH analysis. Generally, rare variants originate from recent mutation, which causes a negative correlation between variant frequency and haplotype length [29]. As expected from this relationship, longer EHH was observed for rare alleles with minor allele frequency (MAF) < 0.1 in bifurcation graphs from our EHH analyses (Table 2); however, we did not analyze these rare alleles further. Without considering this relationship, an apparently longer EHH of a rare allele compared with that of a major allele may produce a misleading inference about the selection of haplotypes with rare alleles.

### 3.5. Effects of Recombination

We investigated the effect of recombination on S* analysis and phylogeny estimation. Our primary aim of S* analysis was to determine haplotypes likely to have been introgressed from archaic hominins to Eurasians. This also exemplified how recombination affects S* analysis and phylogeny estimation. As one example, haplogroup *O* of the HYAL locus contains high-S*-scoring haplotypes, although closely related haplogroups do not. Separately, haplogroup *C* of the same locus contains high-S*-scoring modern humans as well as a haplotype of Altai Neanderthals, suggesting introgression from Altai Neanderthals (Figure 1, Table 3). This distribution of detached high-S*-scoring haplotypes in haplogroup *O* may suggest a trace of recombination with haplogroup *C*. This kind of detached S* distribution in the phylogenetic tree and network was also found for haplogroup *T* in dys44 and haplogroup *N* in OAS.

One alternative explanation of the detached high-S*-scoring haplotypes in the haplotype phylogeny is that a novel combination of two rare alleles in a recombinant haplotype may yield a high S* score without introgression. Because the S* score indicates the possibility of introgression based on two rare alleles colocalized within a haplotype that are absent from the reference population (Africans in this case), recombination may generate novel combined haplotypes that are absent from the reference population.

Not only the S* distribution, but also the allele distribution of a focal SNP in a phylogenetic network can indicate recombination. An example of this was found for OAS. The derived allele distribution in the phylogenetic network shows that haplogroup *F* was located far from other haplotypes bearing derived alleles (gradient blue color tone of Figure 2; Table S1). This suggests recombination between haplotypes bearing ancestral and derived alleles at the focal SNP. Our results on haplotype phylogeny possibly contain more recombinant haplotypes than in other studies because we focused on exact phasing without removing recombinant candidates in this study (see Section 5). Further studies are expected to discriminate recombinants to demonstrate the effects of recombination on phylogeny and to draw conclusions on the relationship between recombination and the detached distribution of characteristic haplotypes in phylogeny.

We also identified the importance of noting that a haplotype is determined based on the positional relationship between recombination and boundaries defining a locus when comparing it with other studies. Our longer-examined genomic region that was uniformly defined using LD values provided a different relationship among landmark haplotypes that had been defined as shorter regions in previous studies. This could be explained by recombination between shorter target regions of each study. Mendez et al. (2012) [23] determined haplotypes of a diversity panel based on the 5′ end region (about 7 kb), which included exons 1–3, whereas in another study, this group (Mendez et al., 2013) [24] started typing based on 15 SNPs that spanned approximately 760 bp at the 3′ end, which included exons 4–6 of the OAS1 gene.

Each study recognized gene genealogical relationships among haplotypes using different landmark haplotypes and estimated introgression candidates. Namely, Mendez et al. (2012) [23] found introgression from Denisovans to Melanesians, while Mendez et al. (2013) [24] used Neanderthals from Vindija Cave and found introgressed haplotypes in Eurasians. Our locus (30.9 kb) overlapped with the 3′ end, including exons 4–6 of the OAS1 gene, and showed that Eurasian haplotypes had a close relationship with a Neanderthal from the Altai Mountains. However, our study did not provide evidence of post-OOA introgression from Denisovans (topological pattern FA; Figure S1; Table 2) as found in the previous study. Further investigation of recombination between the two loci included in Melanesian samples is needed.

### 3.6. Comparison Between Phylogenetic Inferences by Network and Distance Methods

The most intriguing example representing inconsistencies between the phylogenetic tree and network is the MCPH1 locus. This indicates the importance of including a mathematical model to estimate the amount of nucleotide change.

Generally, a phylogenetic tree represents one possible relationship among haplotypes comprehensively shown in a network. Phylogenetic trees are advantageous for representing the relationship of a large number of haplotypes if appropriate mathematical processing can clearly select the most plausible phylogenetic tree. Accordingly, we first constructed an NJ tree using all haplotypes (Figure 1). In contrast, networks can display phylogenetic relationship simultaneously even if recombination, horizontal transfer, and other chromosomal rearrangements have occurred. This prompted us to comprehensively depict phylogenetic relationships in a network as simply as possible. However, a network with a large number of OTUs is apt to be too complex to be understood, in contrast to a tree. Therefore, we used networks to clarify the phylogenetic relationships among clusters shown on the NJ trees by selecting representative haplotypes of the clusters. Furthermore, we used pie charts as OTUs in the network to represent the haplotype frequencies in each cluster except 17q21inv locus (Figure 2).

The incongruence between the phylogenetic tree and the network for the MCPH1 locus illustrated the respective strengths and weaknesses of each method (Table S1). This is shown both in phylogenetic analysis and in EHH analysis. For example, haplotypes bearing a derived allele “*C*”, namely, haplotype *R*, is separate from haplotypes *Q*, *S*, *T*, and *U* in the network (gradient blue color tone of Figure 2IV), despite the clumped distribution in the NJ tree (gradient blue color tone of Figure 1IV). This contradiction can be explained as follows; First, as our EHH analysis indicated, the difference in selection pressure among haplotypes likely produced differences in the length of the region under LD among them (Figure 3, Table 2). Then, the lengths of the haplotypes under LD with beneficial alleles increased because of less frequent recombination than in other haplotypes; this involves a selective sweep, as the EHH of the MCPH1 locus indicated (Figure 3A). Because the recombinant haplotypes carry a mixture of different ancestral information, the numbers of SNP sites that shared ancestry (synapomorphic SNPs) in the examined loci were inconsistent among haplotypes; that is, haplotypes with short regions of EHH shared fewer synapomorphic SNPs than those with long regions of EHH. This might produce systematic error when inferring phylogeny among haplotypes. This inconsistency in evolutionary background among haplotypes results from our definition of loci, which were uniformly determined by their *r*^2^ values. Second, a phylogenetic network based on character-state data can be more vulnerable to inconsistency in the length of regions under LD than distance methods because distance methods include a process of correction of nucleotide substitution based on model studies; this differs from the median network, which is based on the character state without any weighting for the number of synapomorphic SNPs. To exploit the benefits of both phylogenetic trees and networks, a mathematical model to estimate the number of nucleotide changes should be incorporated into the network method, which can represent recombination among the haplotypes examined. These representative loci are expected to be further analyzed to present a comprehensive and powerful model with the full recombination and coalescence process, such as ancestral recombination graph (ARG) based on the sequential Markov coalescent (SMC) model [49].

## 4. Conclusions

Focusing on the eight independent loci that had previously been discussed with regard to introgression from archaic humans for their atypical diversity compared with assumptions of the “out-of-Africa” model, we demonstrated how different loci have diverse evolutionary histories. This has been overlooked because recent genome-wide demographic analyses have tended to focus on detecting population migrations, while locus-specific analysis is apt to detect too much selection. We also showed that introgression from archaic hominin is highly likely when a haplotype of archaic hominin is clustered with multiple modern haplotypes with high S* in phylogenetic trees. However, it should be noted that high S* appeared at the isolated haplotype in phylogeny may have been due to lack of comprehensiveness of reference population and a recent novel recombination brought by a modern African, rather than gene flow with archaic hominins.

## 5. Methods

### 5.1. Data

The data set comprised the genomes of 1092 individuals from 14 populations, including those of European, East Asian, sub-Saharan African, and the American descent [29] (Table S2). The “American” samples used in the 1000 Genomes Project were determined to represent admixture of various North Americans who were more closely related to Africans than to Native Americans [29].

The dataset was constructed using a combination of low-coverage whole-genome and exome sequencing. We downloaded VCF and index (.tbi) files of chromosomes from the ftp site of the 1000 Genomes Project [50] (http://ftp.1000genomes.ebi.ac.uk/vol1/ftp/release/20110521/, accessed between 12 February 2014 and 21 May 2014). The version of the VCF files was Phase 1 Version 3. Phasing for diploid autosomes was conducted in ShapeIt2. The file names include chromosome names and version information, “SHAPEIT2_integrated_phase1_v3.20101123.snps_indels_svs.genotypes.all.vcf.gz” for autosomes, and “phase1_release_v3.20101123.snps_indels_svs.genotypes.all.vcf.gz” for the X chromosome.

### 5.2. Definition of Loci

Eight loci were selected from the literature on the basis of exhibiting unusually diversified haplotypes, as defined by the following criteria: First, the literature on population-based studies indicates that the diversified haplotype is unusually divergent in terms of its sequence and/or its geographic distribution, as postulated by the OOA model. Second, the sequence and genome coordinates of the diversified haplotype were readily discernible based on the descriptions provided in each preceding paper. A manual inspection of the haplotype sequences reported in previous studies was conducted. Based on this inspection, the most diverged haplotype, or the earliest diverged haplotype, was identified as the focal haplotype of interest. In cases where the value of the minor allele count of the focal SNP was insufficient to determine an LD block, a modern human haplotype that matched or was most closely related to Neanderthal sequences was selected as the focal haplotype. A haplotype-specific SNP site that specifically discriminated the focal haplotype (focal SNP) was identified at each locus to determine the LD block.

### 5.3. LD Region Determination

We calculated the *r*^2^ values for all combinations of SNPs that existed within 200 kb in both directions of the ancient haplotype regions using data downloaded from the 1000 Genomes Project; for this, we used VCFtools with the –hap-r2 optional command [51]. We extracted SNPs that were closely associated (i.e., *r*^2^ ≥ 0.8) with the focal SNP. We defined these LD regions as loci to be examined (Table 1).

In the application of our method to the 17q21inv locus, a genomic region with high LD (i.e., *r*^2^ ≥ 0.8) was further examined to clarify the state of duplication within the LD region. The distribution of *r*^2^ values within the LD region over 17q21 was divided into clusters according to a density-based clustering algorithm, Density Reachability and Connectivity Clustering [52], using fpc (v.2.1.6) in the R library (https://CRAN.R-project.org/package=fpc, accessed on 27 January 2014) with the parameters ε = 50,000 and MinPts = 50. On the basis of the results of chr17:43654468–44369518, we eliminated the region that showed duplication and finally obtained chr17:43654468–44205122 as a region for further analysis. Consequently, the defined genomic region did not include the known and intensively focused SD that segregates sub-haplotype H2D in the H2 haplotype and H1D in the H1 haplotype (see Section 3).

### 5.4. Data Validation

Data cleaning and re-genotyping:

The obtained VCF files (http://ftp.1000genomes.ebi.ac.uk/vol1/ftp/release/20110521/, accessed between 12 February 2014 and 21 May 2014) were trimmed according to the definition of the loci [50]. Because the VCF files contained regions with insufficient depth of short-read data, we conducted a preliminary investigation into whether base-calling of the VCF files might be improved by manual comparison using raw data (i.e., base-calling and quality values in BAM files) for five individuals per locus (http://ftp.1000genomes.ebi.ac.uk/vol1/ftp/phase1/data/, accessed between 28 January 2014 and 9 May 2014). This preliminary study showed inconsistency between base-calling in VCF and quality in BAM, and a limit of the base-calling method based on the quality values of read sequences. Although the base and phase information of variant sites in VCF files that were congruent with raw data in BAM files was used for further analysis, we rewrote the VCF files to prioritize our observation of the raw data in the BAM file if there were inconsistencies among data sources.

The BAM files and index files for the target genomic regions were downloaded from the same ftp site for the VCF files mentioned earlier using our in-house programs.

We eliminated information of reads described in the BAM files if there were more than two mismatches to the reference genome within 10 bases by replacing the base-call with “N” and the quality value with “0”. We discarded reads if the “PCR or optical duplicate” flag was set as “ON” in the data description.

We calculated quality values of the variant site described in the VCF files by combining quality values or read sequences obtained from the BAM files according to the algorithm in a program (ConstructAnalysis.py) developed by Brad Chapman. An additional document file shows this in more detail (Additional File S1).

Imputation and re-phasing:

Insertion and deletion (indel) sites in the VCF files, which were re-genotyped as necessary, were separated. Excluding those data, we divided the individual data in VCFs based on whether they contained unphased or missing sites. Then, imputation against missing base-call values was performed for each phased and unphased file followed by re-phasing using Beagle 3.3.2 [53]. Each result of the imputation was incorporated into the VCF file. Here, for heterozygous sites in Beagle output, we compared the phase of the heterozygous site with those of the neighboring three consecutive heterozygous sites. When the phases of these three sites did not match, the phase of the heterozygous site was recorded as “unknown phase”. Otherwise, the phase assumed by Beagle was used for the new VCF file. To reduce such unknown information, we repeated imputation and re-phasing in Beagle using the obtained VCF file. Then, we confirmed that the renewed VCF contained fewer “missing” bases and “unphased” chromosomes (Table S6).

### 5.5. Gene Genealogy Analysis of Haplotypes

We obtained the VCF files for chromosomes of an Altai Neanderthal and a Denisovan from the Max Planck Institute for Evolutionary Anthropology website (http://cdna.eva.mpg.de/neandertal/altai/AltaiNeandertal/VCF/, accessed between 12 February 2014 and 21 May 2014; http://cdna.eva.mpg.de/denisova/VCF/hg19_1000g/, accessed between 12 February 2014 and 21 May 2014). We also obtained chimpanzee sequences for the target genomic regions from the UCSC Table browser (http://genome.ucsc.edu/cgi-bin/hgTables, accessed between 12 February 2014 and 21 May 2014). We present detailed information about preparation of the Neanderthal, Denisovan, and chimpanzee sequences in an additional document (Additional File S1).

NJ tree and bootstrapping:

In the VCF files, we evaluated and corrected base-calling and phasing for modern humans in the 1000 Genomes Project and then combined the data with data of Altai Neanderthal, Denisovan, and chimpanzee into a single VCF file that included indel site information. On the basis of the variant information of the VCF files, nonredundant haplotype sequences were generated after removal of sequence data with 0.5% or more deleted sites in length.

With these haplotype sequence data for each locus, we constructed NJ trees [54] and added bootstrap values using a bash shell script, fasta2trebs.bsh, which automatically executes PHYLIP Dnadist for distance calculation between haplotypes under the F84 model that incorporates different rates of transition/transversion based on probabilities given by Kishino and Hasegawa (1989) [55] and Felsenstein and Churchill (1996) [56]; PHYLIP Neighbor for NJ tree construction; and PHYLIP Seqboot for bootstrapping (using 500 iterations for each locus for this study) [57]. This technique was previously reported [58]. We executed some of the data validation of VCF files and NJ tree construction on the NIG supercomputer at ROIS National Institute of Genetics [59].

Selection of operational taxonomic units for the phylogenetic network:

To clarify the phylogenetic relationships among clusters shown in the NJ trees, we constructed a phylogenetic network with selected operational taxonomic units (OTUs) that represent each cluster of the NJ tree. Therefore, we developed an algorithm to select a small number (14–21 in this study) of OTUs and preferably maintain relationships among clusters of NJ trees without bias and arbitrariness. The algorithm includes the following two steps: First, OTUs were selected that comprehensively and homogeneously maintained their distances from each other. Second, OTUs with extraordinary distances from the root were added to the OTUs selected in the first step. The in-house programs for these steps are available at an open repository (https://github.com/ShimadaMK/HumanDivers/, accessed on 22 May 2022). Briefly, the first program, tree_cluster.pl, determined the candidates of representative clusters and representative OTUs for the clusters. Then, the candidates of representative clusters were removed according to the distance to the neighboring candidate clusters. The removal steps were repeated until the number of candidate clusters reached the upper limit that was previously determined (parameter settings are provided in Table S1). The second program, check_tree.pl, detected OTUs that were particularly far from the root of the NJ trees and added them to the OTUs that represented clusters in the first step. The haplotype sequences without indel sites of these selected OTUs and the two archaic hominins were saved as VCF files.

Construction of the phylogenetic network:

The VCF file was transformed into an RDF file using an in-house perl script. Another VCF file with chimpanzee data was also used to check root position. We constructed a reduced median network [60] with these RDF files using the free software Network 4.6 (https://fluxus-engineering.com/sharenet.htm, accessed on 17 June 2022). When an excessively large number of parallelograms made visualization and interpretation difficult, we adjusted the reduction threshold to reduce unnecessary median vectors and links by manual testing according to the user guide document (parameter settings are provided in Table S1).

### 5.6. S* Analysis

To estimate distinct gene flow events from archaic hominins after OOA, we conducted S* analysis, which was originally devised for analyses with a small number of individuals, such as 20 [26]. Later, Vernot et al. [27,61] extended this approach so that it can be applied to a large number of individuals, and added a step to statistically quantify the matching between a candidate haplotype for introgression and an archaic haplotype. These previous S* calculation methods [26,27] were assumed to use unphased data. Because we used phased data, we modified the original S* method [26] so that it could be applied to phased haplotype data, including missing alleles, and was based on allele distance of a haplotype and not genotype distance on a non-African individual. To avoid obscuring the possibility of introgression from unknown archaic hominins by overemphasizing known archaic hominins, we simply displayed the haplotypes that deviated from the OOA model with classification by the intensity of S* score. We defined the reference population as the African populations LWK, YRI, and ASW (Table S2). We selected a set of SNP sites consisting of ones at which the target chromosome had minor alleles and with African minor frequencies of less than 5%; this was to minimize the possibility of gene flow between non-African (target) and African (reference) populations in the S* calculation. We separately calculated S* for three target populations (European, Asian, and American).

Algorithm:

We largely followed the sequence of steps for S* calculation described in previous reports [26,61]. However, we calculated S* of the target chromosome by defining as the maximum value of summing distances for all subset of SNP pairs that was calculated based on the distance between genomic positions of the two SNP sites and number of chromosomes. We present the detailed algorithm in an additional document (Additional File S1).

Classification and display of S* results on the phylogenetic tree/network:

To simplistically display S* intensities on phylogenetic trees and networks, we classified S* values into three classes at each locus. High and intermediate classes were defined as introgression grades I (*T*2 ≤ S*) and II (*T*1 ≤ S* < *T*2), respectively. We first determined *T*1 and *T*2 at dys44 and RRM2P4 loci by visual observation of the distribution of S* values as *T*1*_dys_*_44_ = 60,000, *T*2*_dys_*_44_ = 80,000, *T*1*_RRM_*_2*P*4_ = 40,000, and *T*2*_RRM_*_2*P*4_ = 53,333. We chose these two loci because they slightly overlap with genic regions. Thresholds for other loci were calculated by assuming a linear relationship between the thresholds and number of SNPs, *N*, in these two loci, dys44 and RRM2P4, as follows:

(1) $$T1=\frac{{T1}_{\mathrm{dys}44}-{T1}_{\mathrm{RRM}2P4}}{N_{\mathrm{dys}44}-N_{\mathrm{RRM}2P4}}N+\frac{{T1}_{\mathrm{RRM}2P4}N_{\mathrm{dys}44}-{T1}_{\mathrm{dys}44}N_{\mathrm{RRM}2P44}}{N_{\mathrm{dys}44}-N_{\mathrm{RRM}2P4}}$$

by assigning *N_dys_*_44_ = 313, *N_RRM_*_2_*_P_*_4_ = 209, and the above-mentioned values.

(2) $$T1=\frac{2500}{13}\left(N-1\right)$$

The second threshold *T*2 was defined as follows:

(3) $$T2=\frac{4}{3}T1$$

### 5.7. EHH

We added ancestral allele information obtained from the UCSC genome browser to the VCF files, and conducted data cleaning and re-imputation in Beagle 3.3.2 [53]. For the “rehh” (v.1) R package [62], we generated two input files (i.e., for haplotypes and SNPs) from the VCF files with an in-house perl program. We set a focal SNP site for EHH analysis as the same SNP site that was used to determine the LD region. If there were multiple focal SNPs with perfect association (i.e., *r*^2^ = 1), a centrally located SNP was chosen. Because we applied the same criteria for choosing focal SNP sites for EHH analyses over all loci, the chosen SNP site was occasionally different from the “SNP for marker of introgressive haplotype” described in the original study, which happened at the HYAL locus; that is, rs116075629 was chosen instead of rs12488302 [25]. We confirmed that the phylogenetic relationship of the HYAL haplotypes obtained in this study was equivalent to that in the original paper published by Ding et al. (2013) [25], and this finding does not change the main argument regarding introgression from Neanderthals. We excluded haplotype data that contained many missing genotype sites by setting the min_perc_geno.hap = 99.999 option of data2haplohh in the rehh program. EHH calculation results were represented in EHH plots. EHH regions were defined as genomic regions with EHH values ≥ 0.05 in both ancestral and derived alleles of the focal SNPs. We also created a bifurcation graph within the regions with EHH values ≥ 0.2. In the case of MAF < 0.1, the obtained results were only used to identify genomic regions with stronger constraints by SNP density differences within the EHH region without comparing alleles to investigate selective sweep. This is because haplotypes with rare SNP variants tend to have less haplotype variation, which elongates regions with EHH in bifurcation graphs irrespective of selection.

## Figures and Tables

**Table 1:** Loci determined by LD region with focal SNPs.

#.	Locus	LD Regions (GRCh37, hg19)				Focal SNPs		Information of Alleles				Prior Knowledge of the Haplotype	Names of Haplotypes		Obs.	Reference
		Chr.	Start	End	Length (bp)	SNP Position	rsID	F.	MAF. (1000G)	Anc./ Der.	V.		Previous Study	Present Study		
I	Xp11hs	Xp11.22	50,521,806	50,604,915	83,110	50,577,285	rs17249510	G	Major	Anc.	0			others	D., N.	[6]
								C	0.0156	Der.	1	Deeply diverged	*hX*	A, B		
II	dys44	Xp21.1	32,226,416	32,261,577	35,162	32,237,621	rs11795471	A	Major	Anc.	0			others		[4,16]
								G	0.0829	Der.	1	Introgressed	*B006*	B, C, T	D., N.	
III	RRM2P4	Xq27.3	143,370,584	143,393,781	23,198	143,393,428	rs6649724	T	Major	Anc.	0			others	D.	[5,17]
								G	0.0787	Der.	1	Introgressed	*Clade A*	A~C	N.	
IV	MCPH1	8p23.1	6,270,149	6,337,231	67,083	6,302,183	rs930557	G	0.3552	Anc.	0	Original		others	D., N.	[18]
								C	Major	Der.	1	Introgressed	*D*	Q~U		
V	17q21inv	17q21.31	43,654,468	44,205,122	550,655	43,856,639	rs62057061 ← rs117245596	G	0.0861	Anc.	1	See discussion	*H2*	A~D		[19,20,21]
								C	Major	Der.	0		*H1*	others	D., N.	
VI	STAT2	12q13.3	56,623,347	56,753,822	130,476	56,750,204	rs2066819	C	Major	Anc.	0			others	D.	[22]
								T	0.0313	Der.	1	Introgressed	*N*	B~E	N.	
VII	OAS	12q24.13	113,350,796	113,381,695	30,900	113,357,442	rs2660	G	0.2123	Anc.	0	Introgressed ^(1)^	*Deep* ^(2)^, *R* ^(3)^	A~E, G	N.	[23,24]
								A	Major	Der.	1			Others ^(4)^	D.	
VIII	HYAL	3p21.31	50,240,131	50,417,061	176,931	50,328,173	rs116075629	T	Major	Anc.	0	Both ^(5)^	All ^(5)^	others	D., N.	[25]
								C	0.005	Der.	1		n/a	A		

(1) Locus VII overlapped with two shorter loci that were previously studied: the 5′ end [23] and 3′ end [24] of the OAS1 gene. Frequent recombination between the two loci may cause confusion between the haplotypes introgressed from Denisovans [23] and Neanderthals [24]. (2) [23]. (3) [24]. (4) Haplotype *F* clustered with haplotypes *A*–*E* but contained the derived “*A*” allele of the focal SNP; conversely, haplotype *G* contained the ancestral “*G*” allele despite its closer relationship with haplotypes *H*–*Q* (Figure 1 and Figure 2). (5) A focal SNP differing from the one in the previous study (rs12488302) was selected; then, both alleles were included in the haplotypes bearing the “*T*” allele at the present focal SNP. Abbreviations: Chr., chromosome band; F., allele on forward strand; MAF., minor allele frequency; Anc., ancestral; Der., derived; V, notation in downloaded VCF; Obs., observed archaic allele in 1000 Genomes; D., Denisovan; N., Neanderthal.

**Table 2:** Summary of results.

#	Locus	Phylogenetic Relationship Among Haplotypes		S* Analysis		EHH Analysis			
		N-D (1)	NJ-Tree Topology Pattern (2)	Grade (3)	Cl (4)	CR (5)	AS (6)	Length [bp] (7)	MAF
I	Xp11hs	+	(**(A,C)**,(A,((N,D),**(A,(A,(A,C)))**)))	I	−	+/−	n/a	24,392	**0.1≥**
II	dys44	−	(D,(E,(C,(N,C))),(E,(C,**(A,(C,(A,E)))**)))	I & II	+	−	n/a	11,048	**0.1≥**
III	RRM2P4	−	(((C,D),**(A,C)**),**(A,(C,N))**)	I & II	+	−	n/a	7433	**0.1≥**
IV	MCPH1	++	(A,((A,E),((A,(E,(N,D))),(C,**(A,C)**))))	II	+	−	++	1927	0.1<
V	17q21inv	++	(C,(C,**((A,(N,D)),E)**))	I & II	−	−	−	2737	0.1<
VI	STAT2	−	(D,((C,N),(E,**(A,C)**)))	I & II	−	−	n/a	18,136	**0.1≥**
VII	OAS	−	((C,(E,N)),(C,(D,**(A,C)**)))	I & II	+	+	−	14,277	0.1<
VIII	HYAL	−	(A,((D,(E,N)),**(A,(C,(A,C)))**))	II	+	+	n/a	11,820	**0.1≥**

(1) Shared the same cluster with Neanderthal (N) and Denisovan (D), (−) not observed; (+) observed with 75% < BS < 99%; (++) observed with BS ≥ 99%. (2) An interpretation of the framework of the NJ-tree topology in Newick format: (A) African; (E) Eurasian; (C) Cosmopolitan; (N) Neanderthal; (D) Denisovan clusters. **Bold** letters indicate typical OOA patterns. (3) Introgression grade observed in multiple haplotypes within a cluster: I for high S* score and II for intermediate S* score indicate high and moderate possibilities of introgression, respectively. See Methods section for details. (4) Colocalization of haplotype with S* and archaic (Neanderthal and/or Denisovan) in the same cluster. (5) Constrained region (CR) was defined by the distribution skewness of SNPs that make a bifurcation in EHH analysis. Shown as observed (+), neutral (+/−), not observed (−). (6) Allelic selection (AS) was defined by EHH range difference between two alleles of the focal SNP at EHH = 0.5 in the EHH plot. Classified by proportion of short to long alleles as (−∞, 0.1), (0.1, 0.2), (0.2, 0.4), and (0.4, 1) for “++”, “+”, “+/−”, and “−”, respectively. Not applied (n/a) if MAF is not more than 0.1. (7) Length of observed high-EHH region (EHH ≥ 0.2).

**Table 3:** Results of S* analysis.

#	Locus	Tree Topological Relationship of Modern Humans		Clustering Pattern of Haplotypes with S*
		To Neanderthal	To Denisovan	
I	Xp11hs	Closely related with an African cluster	Closely related with an African cluster	Except for five African haplotypes used as reference, all haplotypes in the outmost cosmopolitan cluster marked high-grade S*
II	dys44	Clustered with European	External and Independent	Cosmopolitan outer cluster includes high-grade S* and Neanderthal
III	RRM2P4	Clustered with Eurasian	Independent	Cosmopolitan outer cluster includes high-grade S* and Neanderthal
IV	MCPH1	Clustered with S* Asian	Clustered within Asian with S*	Inner cluster includes multiple Asian with medium-grade S*, Neanderthal, and Denisovan
V	17q21inv	Clustered with African	Clustered with African	Outmost cluster includes H2 haplotypes with high or medium-grade S*
VI	STAT2	Independent	Independent	Outer cluster includes multiple Eurasian with S* and located closer to Neanderthal and Denisovan than other modern human clusters
VII	OAS	Clustered with S* Eurasian	Closely related with inner African clusters	Cosmopolitan outer cluster including high-grade S* and Neanderthal
VIII	HYAL	Clustered with Eurasian	Independent	Eurasian cluster *C* includes Neanderthal and multiple medium-grade S*; outmost African cluster A includes one American with high-grade S*

## Data Availability

Computer programs that we created for this study are available at the code hosting platform GitHub, https://github.com/ShimadaMK/HumanDivers (accessed on 22 May 2022).

## Supplementary Materials

The following supporting information can be downloaded at: https://www.mdpi.com/article/10.3390/genes15121554/s1, Figure S1: Expected patterns of haplotype genealogy under models without recombination or contamination; Figure S2: Distribution and maximum of S* values with two determined thresholds; Figure S3: Sequence alignment of SNP sites within the genomic region for EHH bifurcation graph (EHH values ≥ 0.2) in the MCPH1 locus; Figure S4: EHH plots and bifurcation graphs; Figure S5: Correspondence between recombinants from a previous study (Ding et al. 2013) and the current phylogenetic analysis of the HYAL locus; Table S1: Summary of comparison between phylogenetic tree and network for each locus; Table S2: Samples and populations obtained from 1000 Genomes (phase 1 version 3) used in this study; Table S3: Sample origins of haplotypes found in diverged clusters; Table S4: Summary of Samples found in diverged clusters; Table S5: Difference in consequences expected from population models; Table S6: Results of repeated imputation using Xp11hs VCF files; Additional File S1: Detailed information of methods; Additional File S2: Legends of supplementary figures (S1–S5).

## Abbreviations

extended haplotype homozygosity (EHH), incomplete lineage sorting (ILS), insertion and deletion (indel), linkage disequilibrium (LD), minor allele frequency (MAF), million years ago (Mya), neighbor-joining (NJ), out-of-Africa (OOA), operational taxonomic unit (OTU), segmental duplication (SD), single-nucleotide polymorphism (SNP).
