# Supplementary material for: Haplotype-Based Approach Represents Locus Specificity in the Genomic Diversification Process in Humans (Homo sapiens)
Source: Genes (Basel). 2024 Nov 29;15(12):1554. doi: 10.3390/genes15121554 (PMC11675571; doi:10.3390/genes15121554)
Supplement: Supplementary file 1 [file genes-15-01554-s001.zip › Additional File S1.pdf]

## Data validation

Calculation of quality values of the VCF files according to the algorithm in a program (ConstructAnalysis.py) developed by Brad Chapman:

The quality values of a variant site were calculated per site per sequencing platform (ILLUMINA, SOLID, or LS454) per sequencing project (low coverage or exome) as follows:

$$Q = \sum_{k=1}^{n_f} \frac{Qf_k}{k} + \sum_{k=1}^{n_r} \frac{Qr_k}{k}$$

where *nf* is number of reads with forward strands, *nr* is number of reads with reverse strands, $Qf_k$ is the *k*-th largest Phred quality score in reads with forward strands, and $Qr_k$ is the *k*-th largest Phred quality score in reads with reverse strands.

On the basis of our preliminary investigation, we defined the following principles for genotypes according to quality values in BAM files: $Qj$ ($Q1 \geq Q2 \geq Q3 > 0$) for bases with the *j*-th highest quality among bases mapped at the position. First, a base with $Q < 40$ was not assumed to be true (i.e., change to "*N*" for the base). Second, if the ratio in quality values of the two bases mapped on a variant site was not greater than 5, $Q1/Q2 < 5$ ($Q1 \geq Q2$), the site was assumed to be heterozygous. By applying these principles, we rewrote the genotypes in a VCF file using the following criteria:

genotypes re-evaluated for diploid chromosomes =

| | |
|---|---|
| heterozygous (*Q1*/*Q2*) | if $Q2 \geq 40$, $Q1/Q2 < 5$, and $Q2 - Q3 > 5$, |
| heterozygous (*Q1*/*N*) | if $Q2 \geq 40$, $Q1/Q2 < 5$, and $Q2 - Q3 \leq 5$, |
| | or if $Q1 \geq 40$, $Q1/Q2 < 5$, and $Q2 < 40$, |
| homozygous for *Q1* bases (*Q1*/*Q1*) | if $Q1 \geq 40$ and $Q1/Q2 \geq 5$, |
| homozygous for "*N*" (*N*/*N*) | if $Q1 < 40$ |

genotypes re-evaluated for haploid chromosomes =

| | |
|---|---|
| *Q1* | if $Q1 \geq 40$ and $Q1 - Q2 > 5$ |
| *N* | otherwise |

## Gene genealogy analysis of haplotypes

Preparation of Neanderthal and Denisovan sequences:

VCF files for chromosomes of an Altai Neanderthal and Denisovan were downloaded from the Max Planck Institute for Evolutionary Anthropology website (http://cdna.eva.mpg.de/neandertal/altai/AltaiNeandertal/VCF/, http://cdna.eva.mpg.de/denisova/VCF/hg19_1000g/).

After extracting target genomic regions from the chromosomal VCF files, haplotype sequences were determined from the downloaded ancient hominin sequences as follows: (1) bases at sites with "LowQual" in the FILTER field were replaced with alleles of human reference sequences, and (2)

cases other than those described in (1) for which we determined haplotype sequence from diploid data by selecting an allele with a greater read count for single-base substitution sites and selecting reference alleles for indel sites were used.

Preparation of chimpanzee sequence data as an outgroup:
We obtained chimpanzee sequences for the target genomic regions from the UCSC Table browser (http://genome.ucsc.edu/cgi-bin/hgTables) by selecting the following options: clade: Mammal, genome: Human, assembly: Feb. 2009 (GRCh37/hg19), group: Comparative Genomics, track: Conservation, table: Multiz Align (multiz100way), output format: MAF (multiple alignment format).

## S* analysis using phased haplotype data

We conducted S* analysis to estimate distinct gene flow events from archaic hominins after OOA using Africans as a reference population. S* was developed by Plagnol and Wall (Plagnol, V and Wall, JD 2006) and extended by Vernot and Akey (Vernot, B and Akey, JM 2014) for unphased genotype data. Our modification of S* analysis was applied to phased haplotype data. We calculated S* for all chromosomes in three target populations (European, Asian, and American). S* of each chromosome was calculated as described below.
We defined a set of chromosomes and a set of SNP sites for the calculation. The set of chromosomes consisted of 100 randomly selected ones from the same target population containing the target chromosomes. This aimed to uniform the number of samples between populations, considering larger number of samples lead to smaller value of S*. The set of SNP sites consisted of ones at which the target chromosome had minor alleles and with African minor allele frequencies of less than 5%; this was to allow for the possibility of gene flow between non-African (target) and African (reference) populations.
In accordance with the previous studies (Plagnol, V and Wall, JD 2006; Vernot, B and Akey, JM 2014), we scored $R(i,j)$ for pairs of SNP sites $(i,j)$ as follows:

$$R(i,j) = \begin{cases} bp(i,j) + 5000 & (L(i,j) = 0) \\ -10000 & (1 \leq L(i,j) \leq 5) \\ -\infty & (5 < L(i,j)) \end{cases}$$

where $bp(i,j)$ is the distance between genomic positions of the two SNP sites $(i,j)$, and $L(i,j)$ is the number of chromosomes that have the minor allele at one of the two SNP sites $(i,j)$, and have the major allele at the other.
When missing data were included in case of $L(i,j) = 0$, we applied the criteria used in the previous study (Plagnol, V and Wall, JD 2006). Briefly, the set contained no more than two chromosomes with missing calls at an SNP associated with a minor allele of the other SNP in an SNP pair. Moreover, at

most only one such chromosome should exist when the MAF in the set is 2. If the criteria were met for an SNP pair $(i,j)$, then we counted $R(i,j) = bp(i,j) + 5000$; otherwise, it was set as $R(i,j) = 0$. Let a subset of $n$ SNP sites, $J = \{a_1, a_2, \ldots, a_n\}$, where $n \geq 2$ and $bp(a_k, a_{k+1}) \geq 10$. Then,

$$S(J) = \sum_{k=1}^{n-1} R(a_k, a_{k+1}).$$

Finally S* of the target chromosome was defined as the the maximum value of $S(J)$ for all $J$. It was not defined if the set contained less than two SNP sites or in case of $bp(i,j) < 10$ for all $i$ and $j$. Because it would take enormouse computation to calculate all $S(J)$, we used "dynamic programming" that reduced the computational quantity during the search for the maximum value of $S(J)$, in accordance with previous studies (Plagnol, V and Wall, JD 2006; Vernot, B and Akey, JM 2014).

The in-house programs (sstar.pl, sstar2mega_group.pl) for these steps are available at an open repository, github.

(1) S* calculation using sstar.pl

$ sstar.pl --vcf 1000g.vcf --pop test_pop.id --ref ref.hap --size 100 --ref-mac 24 > s-star.txt

Here, as an example of autosomal loci using the 1000 Genomes data, "100" and "24" are the sample size (number of chromosomes to be selected from a target population) and maximum limit of frequency of (less than 5%) a minor allele in the reference population, respectively.

1000g.vcf: a VCF file of the genomic region for which S* is to be calculated,

test_pop.id: list of individuals of a target population for which S* is to be calculated,

ref.hap: list of chromosomes in a reference population that are named by combining individual ID and "-1" or "-2" for each chromosome,

s-star.txt: output file containing S* of chromosomes of individuals listed and their SNP combinations.

(2) Display of S* results in the phylogenetic tree using sstar2mega_group.pl

The sstar2mega_group.pl outputs a group definition file to display using MEGA software.

$ sstar2mega_group.pl -S s-star.txt -G group.txt -C 0,5000,20000,50000,100000 > s-star.mega.txt

Here,

s-star.txt: output file of the sstar.pl mentioned above,

group.txt: list of haplogroup and its chromosome members, as shown, a chromosome name and its group name in two columns separated by a tab per row,

"0,5000,20000,50000,100000" indicates S* values of boundaries of classes,

As a result of the command execution, an output file, s-star.mega.txt, can be obtained that includes the class or group definition based on the S* values in the s-star.txt, namely, "-∞," "-10000," "0," "5000 to 19999," "20000 to 49999," "50000 to 99999," and "100000 or more."

This s-star.mega.txt file can be used for displaying subtree grouping in MEGA software, as follows: “File > Import Group Names” and “Subtree > Draw Options.”
We omitted, -G option in the 17q21inv locus where no OTUs to be changed their names.

## Investigation of removed haplotype in previous study of HYAL locus

To evaluate the impact of recombination on phylogenetic analysis, we focused on haplotypes removed in a previous study (Ding et al. 2013) as recombinants between introgressed and non-introgressed segments. In the previous study, derived alleles of 26 specific SNPs (Type 2 SNPs) were considered to be introgressive haplotypes, and 19 haplotypes bearing both derived (introgressive) and ancestral (non-introgressive) alleles among the 26 Type 2 SNPs were considered recombinants and were removed. In the current study, haplotypes of the HYAL locus were determined in a different way (i.e., in locus definition and phase determination) from that in the previous study, and recombinant candidates were not removed. Then, the correspondence between recombinants from the previous study and the current study was determined.
We investigated the sample ID of individuals with recombinant haplotypes in the previous study. Among the haplotypes obtained from these samples, we compared the obtained haplotypes in the two studies and determined the correspondence between them.
Haplotypes from the current study are shown below and corresponding haplotypes from the previous study are indicated in parentheses.
NA18572-1 (NA18572 b)
NA18609-2 (NA18609 a)
NA18641-1 (NA18641 b)
HG00501-1 (HG00501 a)
HG00590-1 (HG00590 b)
HG00634-2 (HG00634 b)
HG00662-2 (HG00662 b)
HG00692-1 (HG00692 a)
NA18950-1 (NA18950 a)
NA18950-2 (NA18950 b)
NA18968-1 (NA18968 b)
NA18977-1 (NA18977 a)
NA18995-1 (NA18995 b)
NA19735-2 (NA19735 a)
HG01271-2 (HG01271 b)

Haplotypes with incomplete correspondence with candidates in the previous study

HG00651-1 (HG00651 a)
NA18945-2 (NA18945 a)
HG00701-2 (HG00701 a)
HG00436-2 (HG00636 a, possibly a typo of HG00436 a)

Next, we investigated alleles of all SNPs of the haplotypes in the current study and classified whether the haplotypes corresponded to the haplotypes as recombinants in the previous study (closed diamond in Fig. s5) or not (open circle in Fig. s5). These haplotypes were subdivided in terms of recombination between ancestral and derived genomic segments according to alleles of Type 2 (Fig. s5).
