# Supplementary material for: Haplotype-Based Approach Represents Locus Specificity in the Genomic Diversification Process in Humans (Homo sapiens)
Source: Genes (Basel). 2024 Nov 29;15(12):1554. doi: 10.3390/genes15121554 (PMC11675571; doi:10.3390/genes15121554)
Supplement: Supplementary file 1 [file genes-15-01554-s001.zip › Additional File S2.pdf]

Supplementary Figure s1:

Expected patterns of haplotype genealogy under models without recombination or contamination. A: African cluster, E: Eurasian cluster, †: known archaic haplotype found in Eurasia, φ: unknown archaic haplotype.

Type Fo: Recent out-of-Africa (OOA) without incomplete lineage sorting (ILS); type FE: introgression from known archaic hominins to Eurasians after recent OOA and ILS; type FA: introgression from known archaic hominins to Africa, and ancient polymorphisms within Africa; type Af: introgression from ancestral Eurasians to known archaic hominins, and subdivision within Africa before OOA for both archaic hominins and modern humans (cf., Green et al. 2010, Fig. 6); type Ea: introgression from unknown archaic hominins to Eurasians; type Co: introgression from unknown archaic hominins to an ancestral population prior to OOA, and ancient polymorphism within Africa before OOA for both archaic hominins and modern humans, followed by ILS.

Supplementary Figure s2:

Distribution and maximum of S* values with two determined thresholds. Positive S* values were classified into four intervals whose endpoints are written in E-notation to avoid superscript use. Specifically, *m*e+*n* indicates a value of $m \times 10^n$. See Materials and Methods for the formula for calculating S* and determination of the two thresholds.

Supplementary Figure s3:

Sequence alignment of SNP sites within the genomic region for EHH bifurcation graph (EHH values ≥ 0.2) in the MCPH1 locus: 20 haplotypes of 1000 Genomes, and Denisovan and Neanderthal haplotypes that were selected for phylogenetic network construction are displayed, although EHH analysis was conducted with all sequence data of 1000 Genomes. Yellow background indicates extended haplotype regions carrying the ancestral allele (G) at the focal SNP (rs930557) that shown in bold. Haplogroups are shown in square brackets.

Supplementary Figure s4:

EHH plots and bifurcation graphs. (A) Xp11hs, (B) dys44, (C) RRM2P4, (D) 17q21inv, (E) STAT2, (F) OAS, (G) HYAL. See Figure 3 for the MCPH1 locus and detailed information about these graphs.

Supplementary Figure s5:

Correspondence between recombinants from a previous study (Ding et al. 2013) and the current phylogenetic analysis of the HYAL locus was determined. On the basis of all SNPs of the HYAL locus in the current study, haplotypes from the samples with recombinant haplotypes in the previous study

were classified in terms of whether they corresponded to haplotypes as recombinants in the previous study (closed diamond) or not (open circle). Colors indicate the status of haplotypes in the current study regarding recombination between derived (or introgressed from Neanderthal) and ancestral (non-introgressed) genomic segments based on 26 SNPs used to infer introgression in the previous study: such recombination was observed (brown), no recombination was observed but derived alleles were observed (green), and no recombination was observed but derived alleles were observed (red). See additional document (Additional file 7) for details.
