# Supplementary material for: Haplotype-Based Approach Represents Locus Specificity in the Genomic Diversification Process in Humans (Homo sapiens)
Source: Genes (Basel). 2024 Nov 29;15(12):1554. doi: 10.3390/genes15121554 (PMC11675571; doi:10.3390/genes15121554)
Supplement: Supplementary file 1 [file genes-15-01554-s001.zip › FigsN/Fig_s3_EHHbifRegnNet1kgArcID_seq_v2.pdf]

rsID	rs2440416	rs189522330	rs75666535	rs182226349	rs111137028	rs185966191	rs62496812	rs142720060	rs147324953	rs180825999	rs201947779	rs34121009	rs141024274	rs149813931	rs201231900	rs2083914	rs200406468	**rs930557**	rs185613641	rs115088000	rs199750676	rs145820898	rs148526209
chr8(hg19)	6301472	6301481	6301519	6301540	6301546	6301603	6301738	6301859	6301898	6301929	6301980	6302033	6302062	6302102	6302110	6302154	6302178	**6302183**	6302221	6302232	6302279	6302295	6302304
HG00114R [U]	G	A	T	T	G	G	T	A	C	G	A	A	T	A	G	G	C	C	T	A	C	G	T
HG00422R [O]	G	A	T	T	G	G	T	A	C	G	A	A	T	A	G	G	C	G	T	A	C	G	T
HG00740R [K]	C	A	T	T	G	G	T	A	C	G	A	A	T	A	G	G	C	G	T	A	C	G	T
NA18522R [M]	C	A	T	T	G	G	T	A	C	G	A	A	T	A	G	G	C	G	T	A	C	G	T
NA18868R [D]	C	A	T	T	A	G	T	A	C	G	A	A	T	A	G	G	C	G	T	A	C	G	T
NA18870L [F]	C	A	T	T	A	G	T	A	C	G	A	A	T	A	G	G	C	G	T	A	C	G	T
NA18924L [H]	C	A	T	T	A	G	T	A	C	G	A	A	T	A	G	G	C	G	T	A	C	G	T
NA18940R [Q]	G	A	T	T	G	G	T	A	C	G	A	A	T	A	G	G	C	C	T	A	C	G	T
NA18962R [E]	C	A	T	T	A	G	T	A	C	G	A	A	T	A	G	G	C	G	T	A	C	G	T
NA19347L [T]	G	A	T	T	G	G	T	A	C	G	A	A	T	A	G	G	C	C	T	A	C	G	T
NA19372L [S]	G	A	T	T	G	G	T	A	C	G	A	A	T	A	G	G	C	C	T	A	C	G	T
NA19383R [A]	C	A	T	T	G	G	T	A	C	G	A	A	T	A	G	G	C	G	T	A	C	G	T
NA19397R [L]	C	A	T	T	G	G	T	A	C	G	A	A	T	A	G	G	C	G	T	A	C	G	T
NA19435R [B]	C	A	T	T	G	G	T	A	C	G	A	A	T	A	G	G	C	G	T	A	C	G	T
NA19467R [J]	C	A	T	T	G	G	T	A	C	G	A	A	T	A	G	G	C	G	T	A	C	G	T
NA19704R [I]	G	A	T	T	G	G	T	A	C	G	A	A	T	A	G	G	C	G	T	A	C	G	T
NA20336L [C]	C	A	T	T	G	G	T	A	C	G	A	A	T	A	G	T	C	G	T	A	C	G	T
NA20336R [G]	C	A	T	T	A	G	T	A	C	G	A	A	T	A	G	G	C	G	T	A	C	G	T
NA20509L [R]	C	A	T	T	G	G	T	A	C	G	A	A	T	A	G	G	C	C	T	A	C	G	T
NA20582R [N]	C	A	T	T	A	G	T	A	C	G	A	A	T	A	G	G	C	G	T	A	C	G	T
Denisova	G	A	T	T	G	G	T	A	C	G	A	A	T	A	G	G	C	G	T	A	C	G	T
AltaiNeandrtl	G	A	T	T	G	G	T	A	C	G	A	A	T	A	G	G	C	G	T	A	C	G	T

rsID	rs190026099	rs201084851	rs200513127	rs2515569	rs142858644	rs199724219	rs77959215	rs201039834	rs2920676	rs185736569	rs192003514	rs183880522	rs146586991	rs188726432	rs141218500	rs35551093	rs41313954	rs17076894	rs146744659	rs192777656	rs182472674	rs187245785	rs140402469
chr8(hg19)	6302343	6302344	6302391	6302418	6302479	6302487	6302592	6302612	6302671	6302686	6302701	6302723	6302738	6302907	6302959	6302962	6302971	6302981	6303028	6303050	6303140	6303265	6303398
HG00114R [U]	C	G	C	G	T	A	A	G	C	C	A	G	G	C	C	C	C	A	T	C	A	C	G
HG00422R [O]	C	G	C	G	T	A	A	G	C	C	A	G	G	C	C	C	C	A	T	C	A	C	G
HG00740R [K]	C	G	C	G	T	A	A	G	C	C	A	G	G	C	C	C	C	A	T	C	A	C	G
NA18522R [M]	C	G	C	G	T	A	A	G	C	C	A	G	G	C	C	C	C	A	T	C	A	C	G
NA18868R [D]	C	G	C	G	T	A	A	G	T	C	A	G	G	C	C	C	T	A	T	C	A	C	G
NA18870L [F]	C	G	C	G	T	A	A	G	T	C	A	G	G	C	C	C	C	A	T	C	A	C	G
NA18924L [H]	C	G	C	G	C	A	A	G	T	C	A	G	A	C	C	C	C	A	T	C	A	C	G
NA18940R [Q]	C	G	C	G	T	A	A	G	C	C	A	G	G	C	C	C	C	A	T	C	A	C	G
NA18962R [E]	C	G	C	G	T	A	A	G	T	C	A	G	G	C	C	C	T	A	T	C	A	C	G
NA19347L [T]	C	G	C	G	T	A	A	G	C	C	A	G	G	C	C	C	C	A	T	C	A	C	G
NA19372L [S]	C	G	C	G	T	A	A	G	C	C	A	G	G	C	C	C	C	A	T	C	A	C	G
NA19383R [A]	C	G	C	G	T	A	A	G	C	C	A	G	G	C	C	T	C	A	T	C	A	C	G
NA19397R [L]	C	G	C	G	T	A	A	G	C	C	A	G	G	C	C	C	C	A	T	C	A	C	G
NA19435R [B]	C	G	C	G	T	A	A	G	C	C	A	G	G	C	C	T	C	A	T	C	A	C	G
NA19467R [J]	C	G	C	G	T	A	A	G	C	C	A	G	G	C	C	C	C	A	T	C	A	C	G
NA19704R [I]	C	G	C	G	T	A	A	G	C	C	A	G	G	C	C	C	C	A	T	C	A	C	G
NA20336L [C]	C	G	C	G	T	A	A	G	C	C	A	G	G	C	C	C	C	A	T	C	A	C	G
NA20336R [G]	C	G	C	G	T	A	A	G	T	C	A	G	G	C	C	C	C	G	T	C	A	C	G
NA20509L [R]	C	G	C	G	T	A	A	G	C	C	A	G	G	C	C	C	C	A	T	C	A	C	G
NA20582R [N]	C	G	C	G	T	A	A	G	T	C	A	G	G	C	C	C	C	A	T	C	A	C	G
Denisova	C	G	C	G	T	A	A	G	C	C	A	G	G	C	C	C	C	A	T	C	A	C	G
AltaiNeandrtl	C	G	C	G	T	A	A	G	C	C	A	G	G	C	C	C	C	A	T	C	A	C	G
