# Supplementary figures and images for: Haplotype-Based Approach Represents Locus Specificity in the Genomic Diversification Process in Humans (Homo sapiens)
Source: Genes (Basel). 2024 Nov 29;15(12):1554. doi: 10.3390/genes15121554 (PMC11675571; doi:10.3390/genes15121554)

Fo

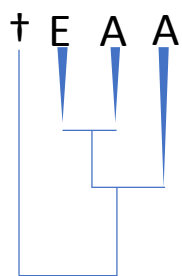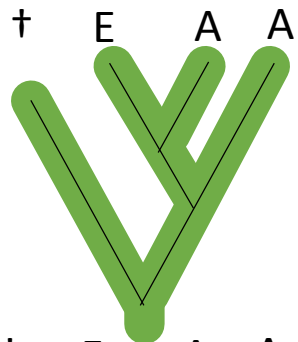

FE

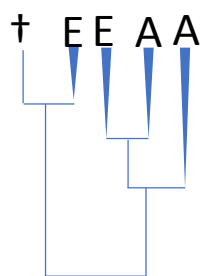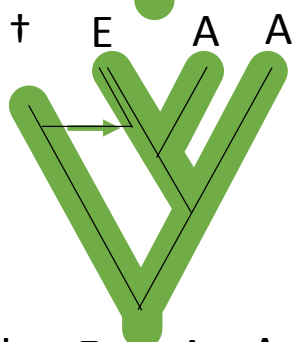

FA

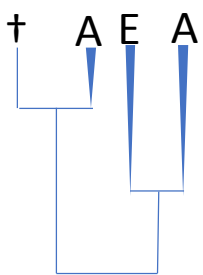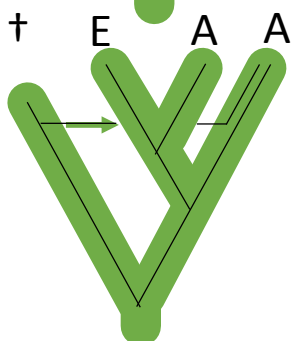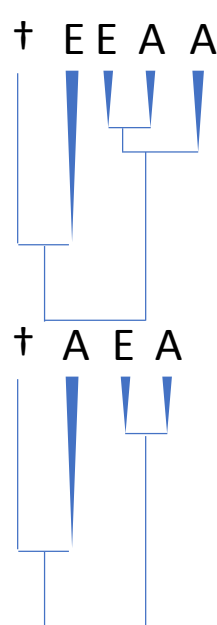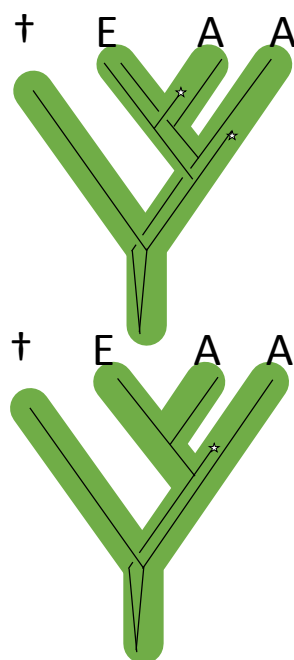

Af

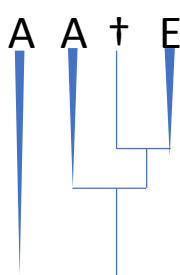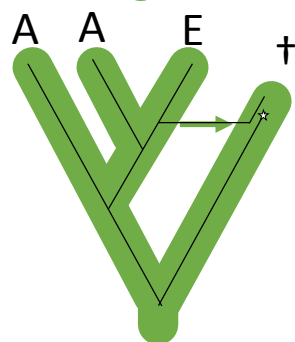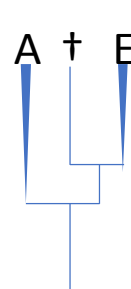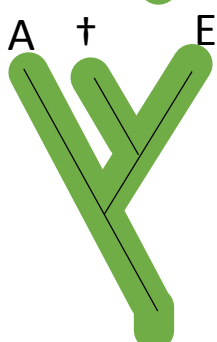

Ea

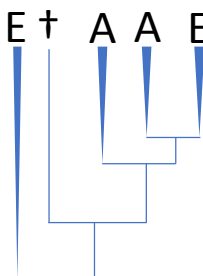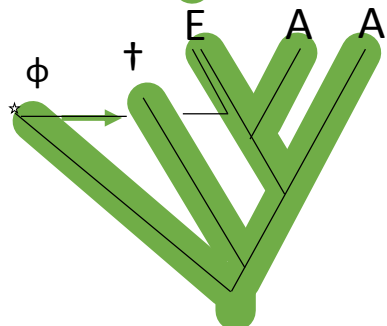

Co

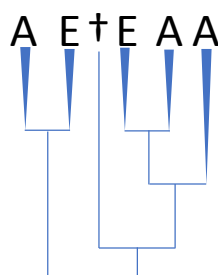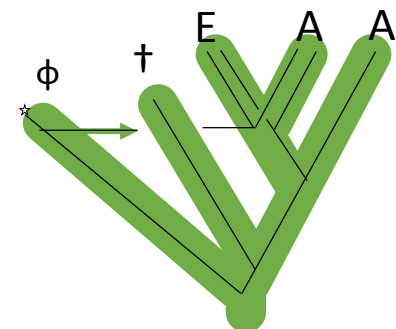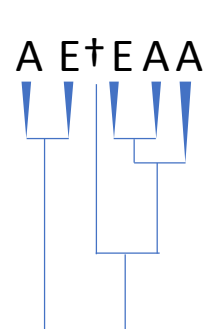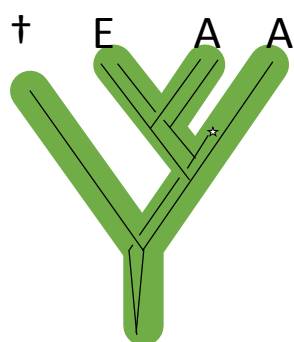

Supplement: Supplementary file 1 [file genes-15-01554-s001.zip › FigsN/Fig_s1_TreeType.pdf]

Xp11hs

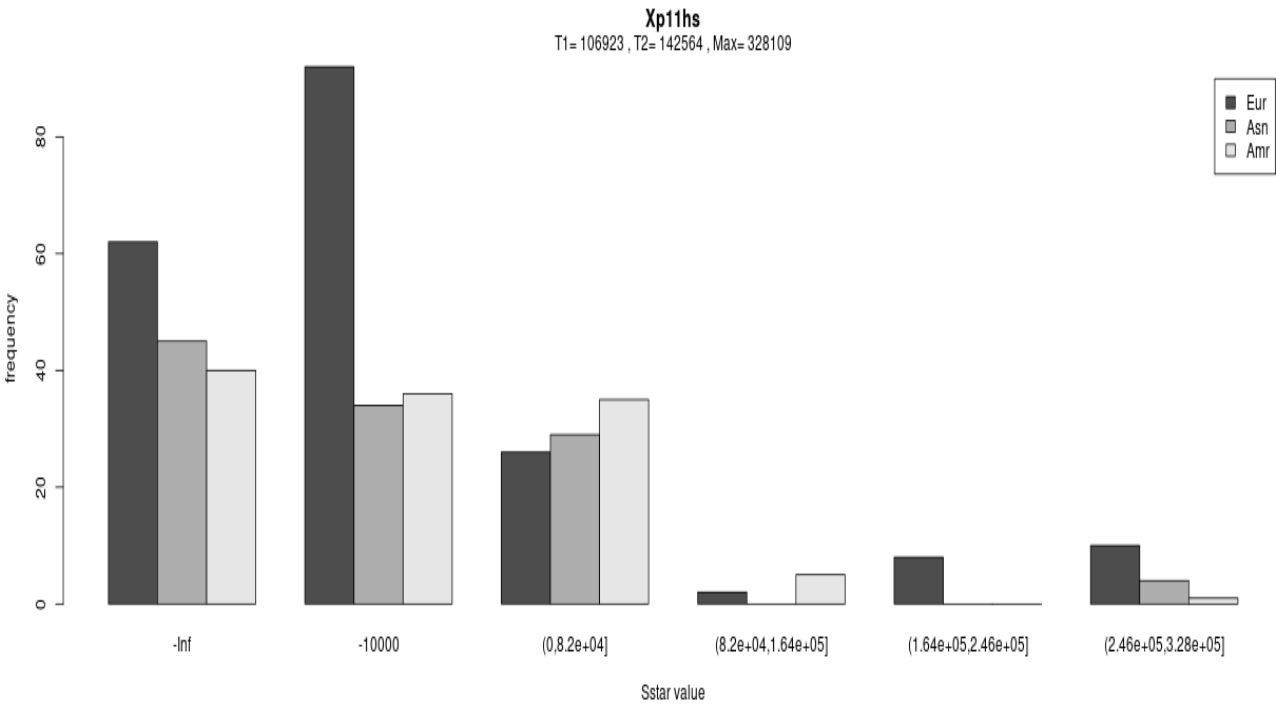

dys44

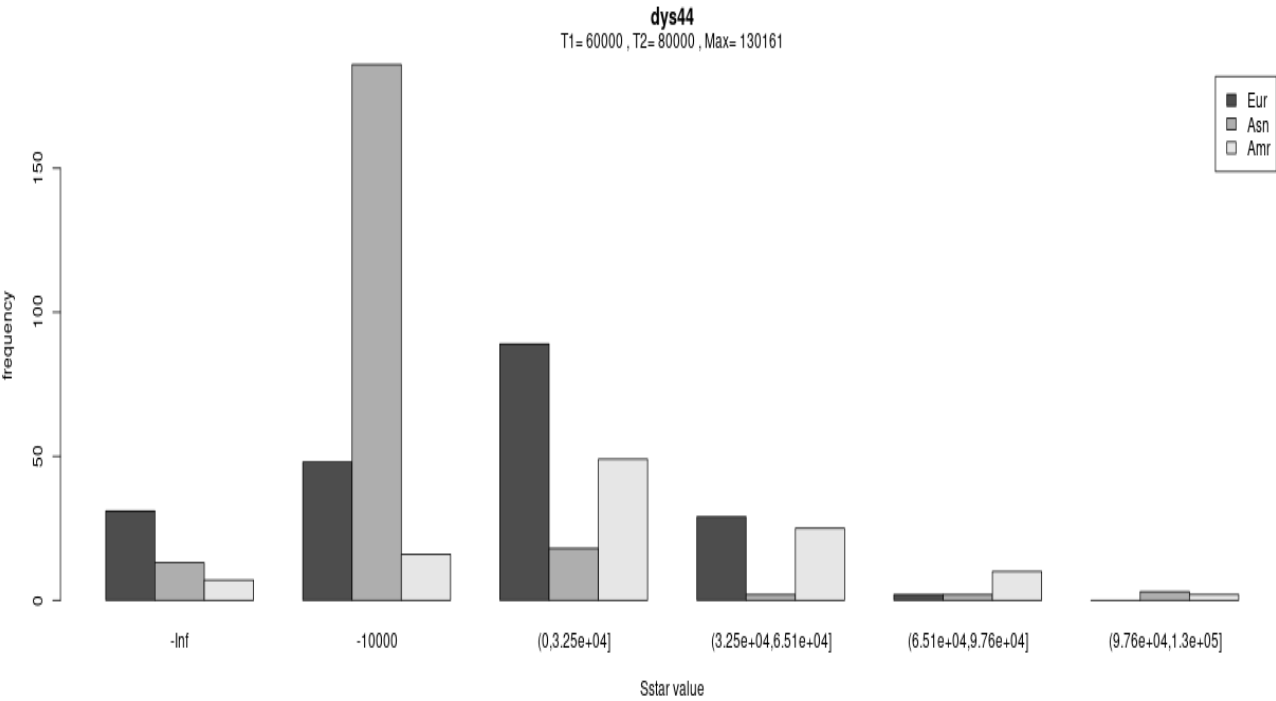

RRM2P4

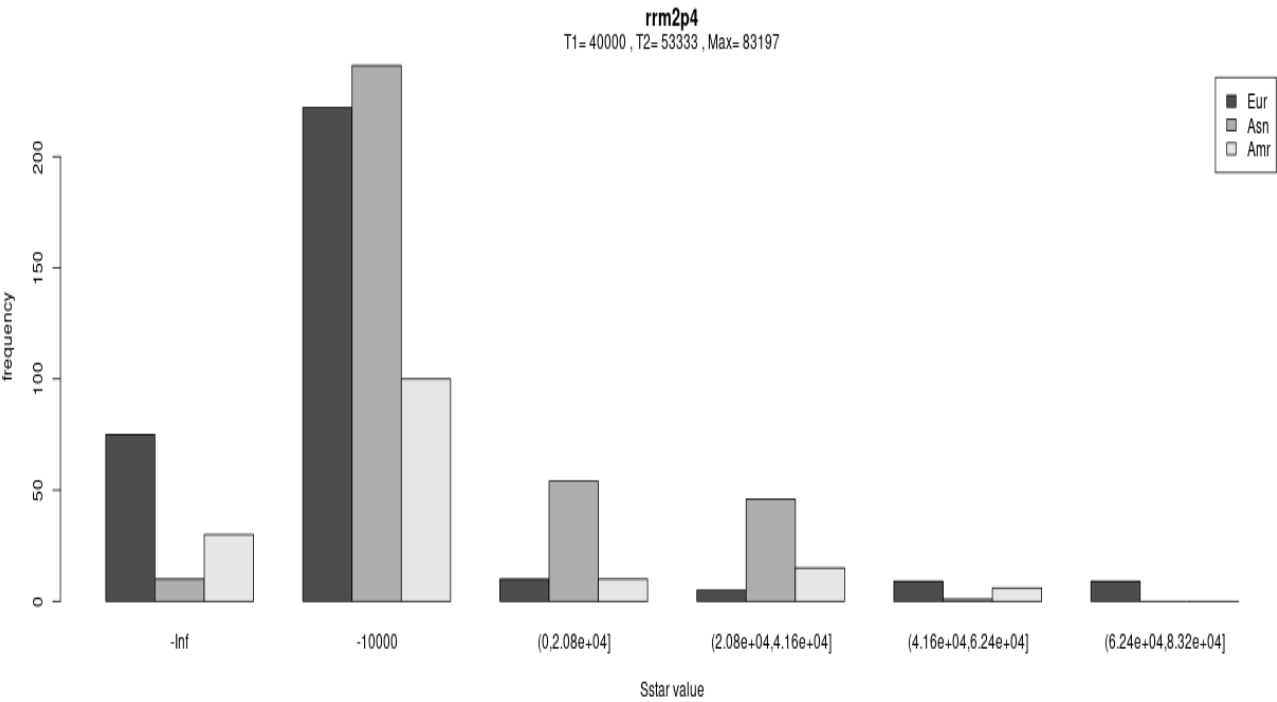

MCPH1

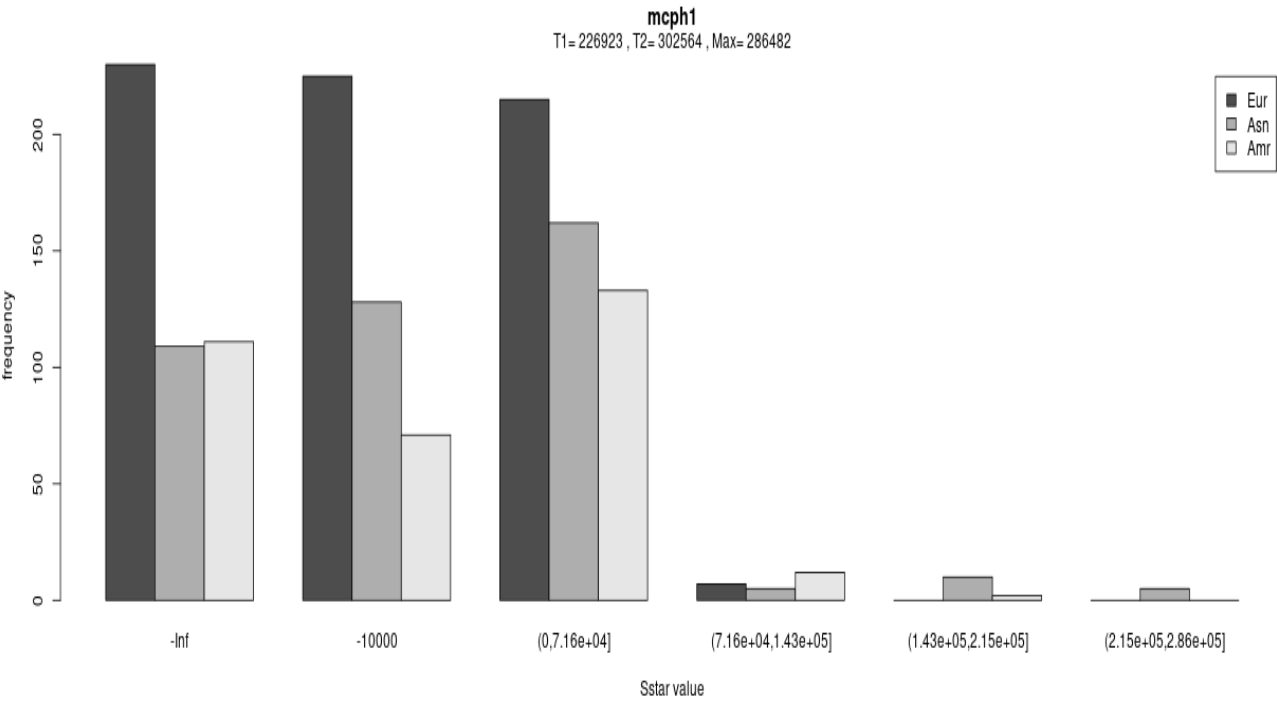

17q21inv

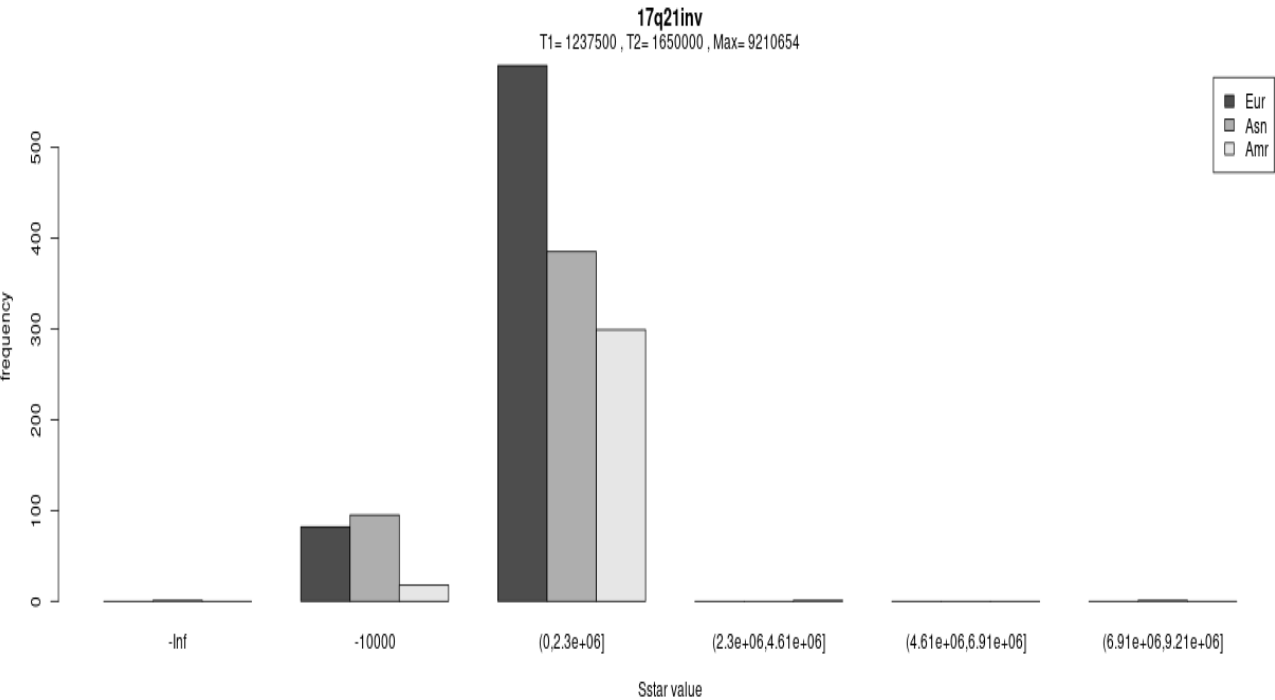

STAT2

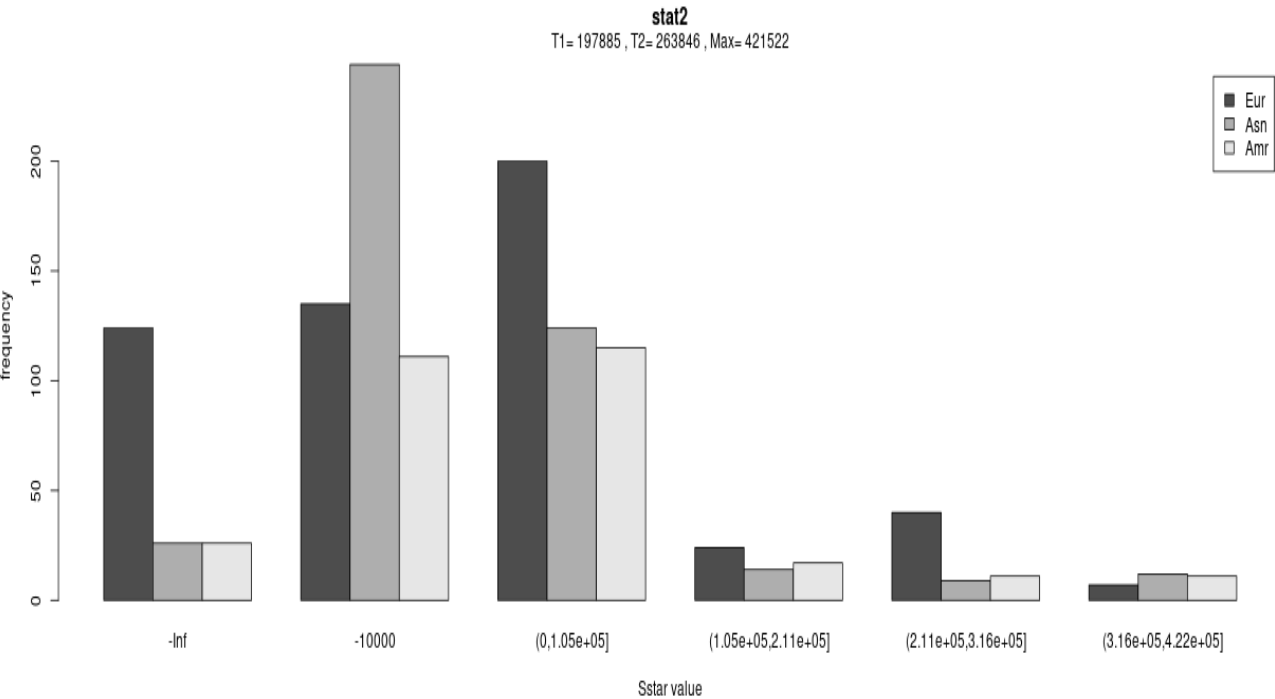

OAS

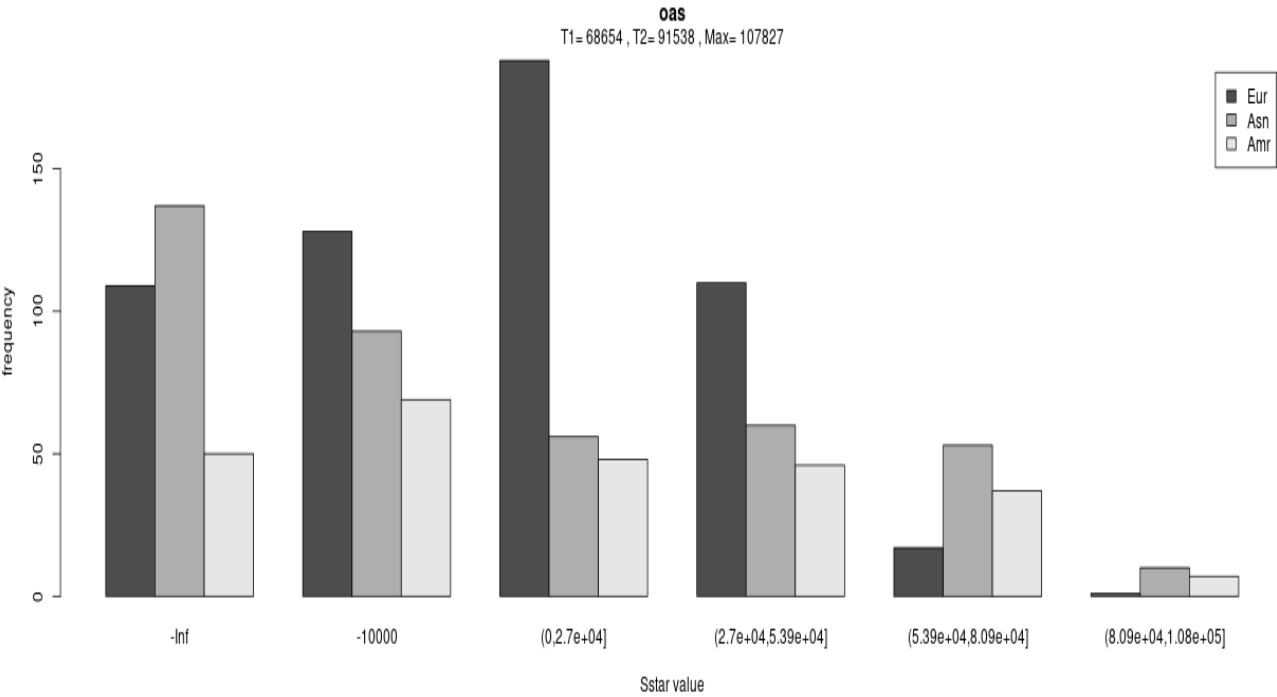

HYAL

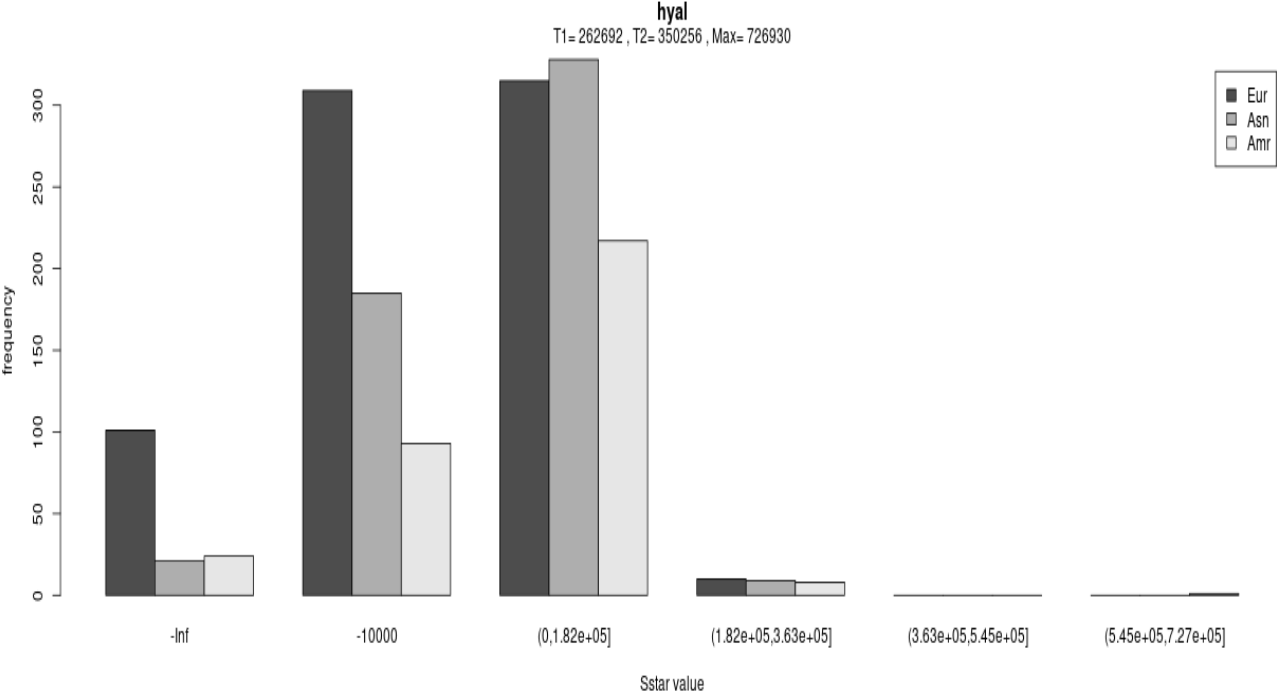

Supplement: Supplementary file 1 [file genes-15-01554-s001.zip › FigsN/Fig_s2_Sstar8lociA4Landscape.pdf]

Fig. s4  
Xp11hs

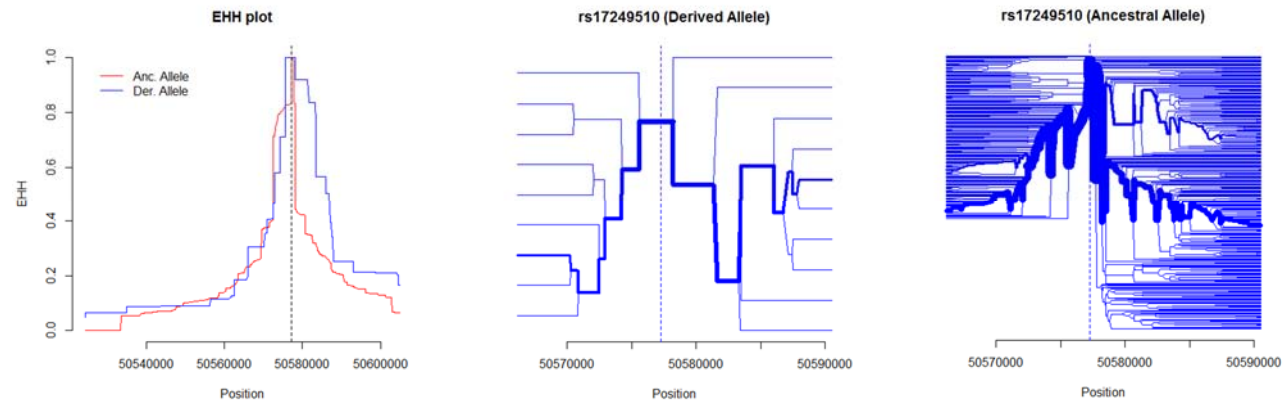

dys44

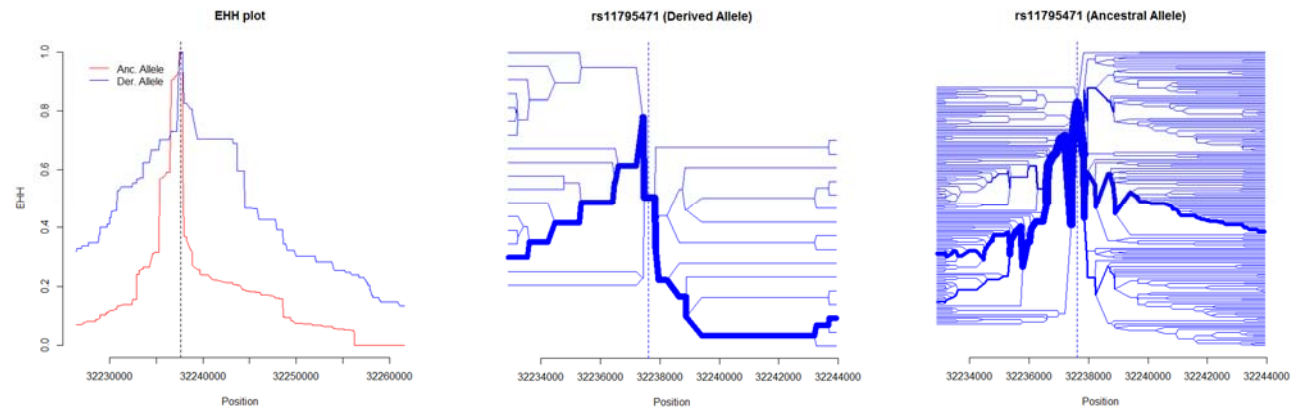

RRM2P4

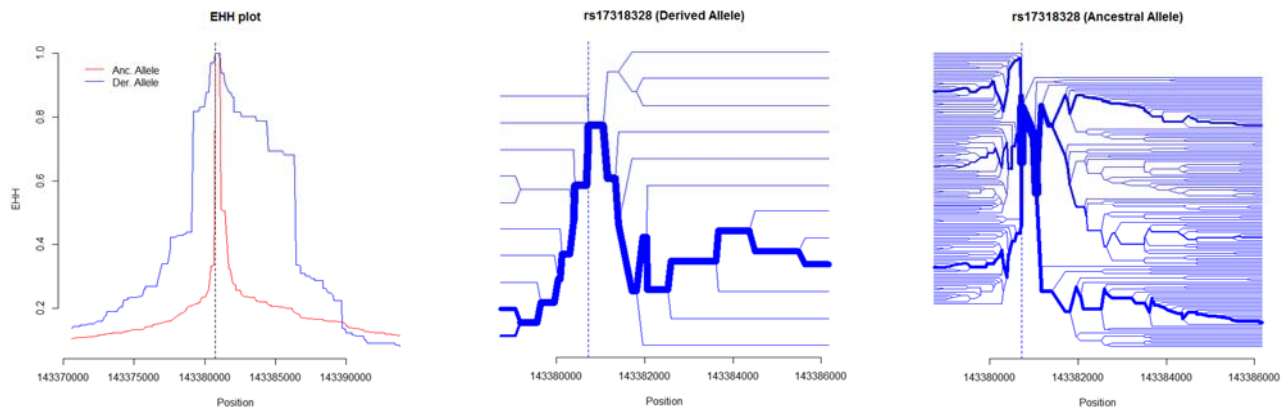

17q21inv

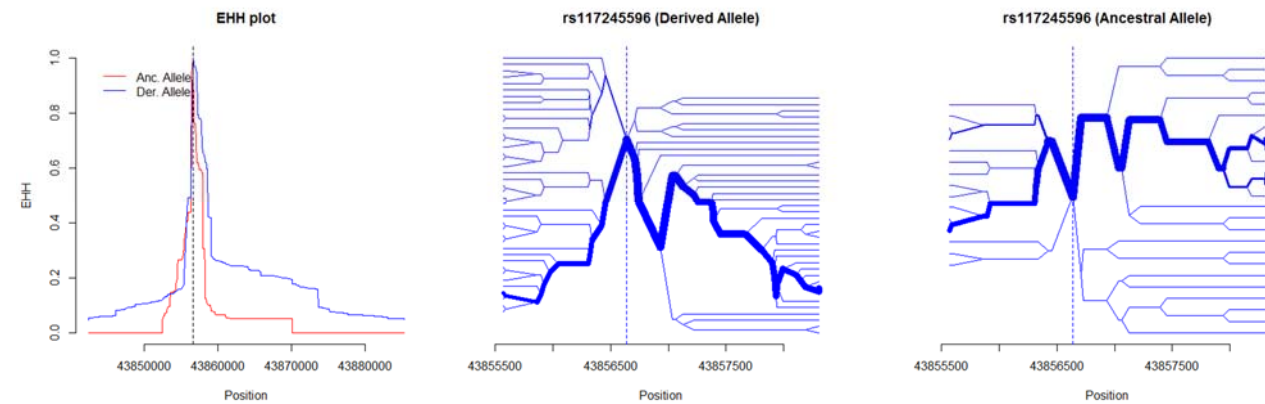

STAT2

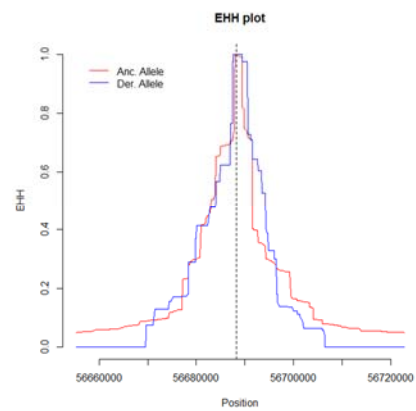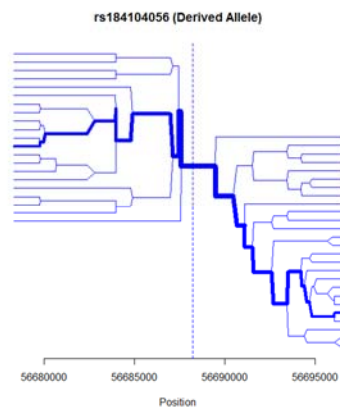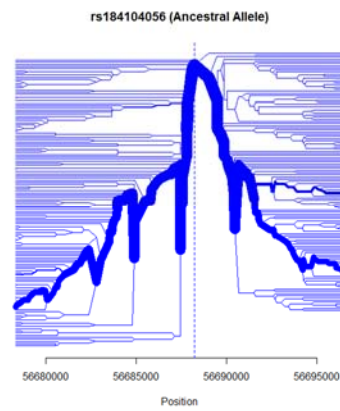

OAS

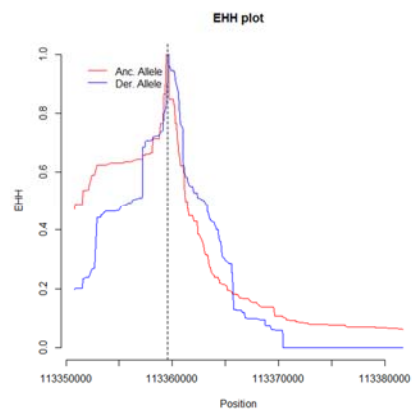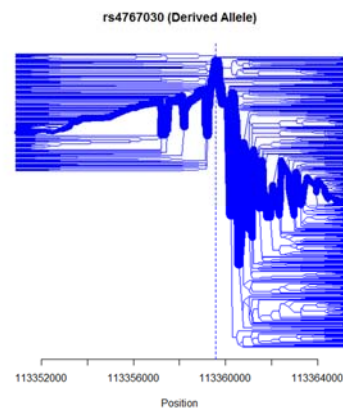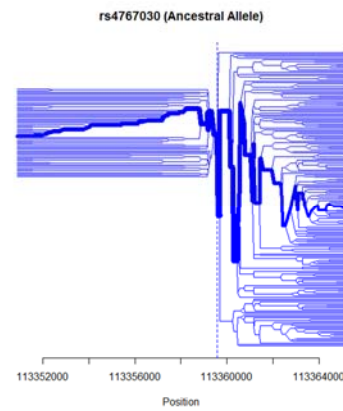

HYAL

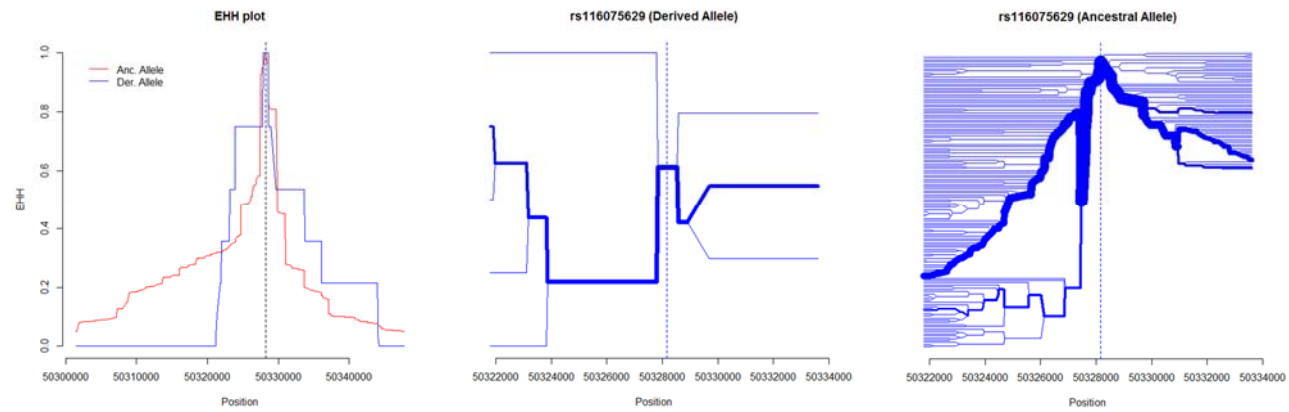

Supplement: Supplementary file 1 [file genes-15-01554-s001.zip › FigsN/Fig_s4_EHHgraphs_supA4Landscape.pdf]

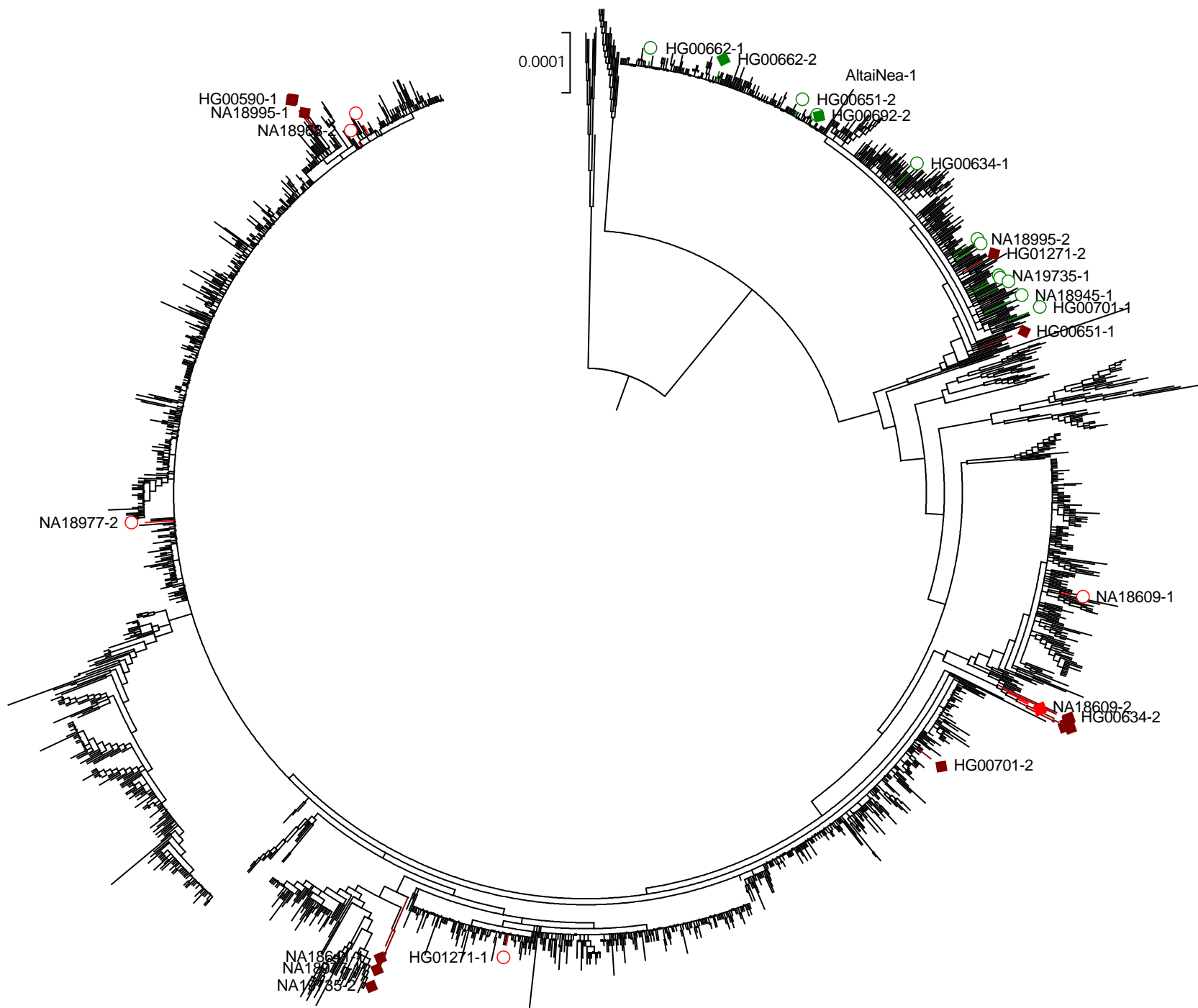

Supplement: Supplementary file 1 [file genes-15-01554-s001.zip › FigsN/Fig_s5_hyal_Type2.recom.pdf]
